# Dynamics of Reactive Carbonyl Species in Pea Root Nodules in Response to Polyethylene Glycol (PEG)-Induced Osmotic Stress

**DOI:** 10.3390/ijms23052726

**Published:** 2022-03-01

**Authors:** Alena Soboleva, Nadezhda Frolova, Kseniia Bureiko, Julia Shumilina, Gerd U. Balcke, Vladimir A. Zhukov, Igor A. Tikhonovich, Andrej Frolov

**Affiliations:** 1 Department of Bioorganic Chemistry, Leibniz Institute of Plant Biochemistry, D-06120 Halle (Saale), Germany or kbureiko@uef.fi (K.B.); st014297@student.spbu.ru (J.S.); 2 Department of Biochemistry, St. Petersburg State University, 199034 Saint Petersburg, Russia; 3 Department of Plant Physiology and Biochemistry, St. Petersburg State University, 199034 Saint Petersburg, Russia; st806497@spbu.ru; 4 Institute of Biomedicine, University of Eastern Finland, FI-70211 Kuopio, Finland; 5 Department of Metabolic and Cell Biology, Leibniz Institute of Plant Biochemistry, D-06120 Halle (Saale), Germany; gerd.balcke@ipb-halle.de; 6 All-Russia Research Institute for Agricultural Microbiology, Podbelsky Chaussee 3, Pushkin 8, 196608 St. Petersburg, Russia; vladimir.zhukoff@gmail.com (V.A.Z.); or contact@arriam.spb.ru (I.A.T.); 7 Department of Genetics and Biotechnology, St. Petersburg State University, 199034 Saint Petersburg, Russia

**Keywords:** 7-(diethylamino)coumarin-3-carbohydrazide (CHH), derivative stability, 4,5-dioxovaleric acid, drought, legume-rhizobial symbiosis, metabolomics, osmotic stress, pea (*Pisum sativum* L.), reactive carbonyl compounds (RCCs), root nodules

## Abstract

Drought dramatically affects crop productivity worldwide. For legumes this effect is especially pronounced, as their symbiotic association with rhizobia is highly-sensitive to dehydration. This might be attributed to the oxidative stress, which ultimately accompanies plants’ response to water deficit. Indeed, enhanced formation of reactive oxygen species in root nodules might result in up-regulation of lipid peroxidation and overproduction of reactive carbonyl compounds (RCCs), which readily modify biomolecules and disrupt cell functions. Thus, the knowledge of the nodule carbonyl metabolome dynamics is critically important for understanding the drought-related losses of nitrogen fixation efficiency and plant productivity. Therefore, here we provide, to the best of our knowledge, for the first time a comprehensive overview of the pea root nodule carbonyl metabolome and address its alterations in response to polyethylene glycol-induced osmotic stress as the first step to examine the changes of RCC patterns in drought treated plants. RCCs were extracted from the nodules and derivatized with 7-(diethylamino)coumarin-3-carbohydrazide (CHH). The relative quantification of CHH-derivatives by liquid chromatography-high resolution mass spectrometry with a post-run correction for derivative stability revealed in total 194 features with intensities above 1 × 10^5^ counts, 19 of which were down- and three were upregulated. The upregulation of glyceraldehyde could accompany non-enzymatic conversion of glyceraldehyde-3-phosphate to methylglyoxal. The accumulation of 4,5-dioxovaleric acid could be the reason for down-regulation of porphyrin metabolism, suppression of leghemoglobin synthesis, inhibition of nitrogenase and degradation of legume-rhizobial symbiosis in response to polyethylene glycol (PEG)-induced osmotic stress effect. This effect needs to be confirmed with soil-based drought models.

## 1. Introduction

In the context of oncoming climate changes, water deficit represents one of the major abiotic constraints [1]. Thereby, drought and underlying it osmotic stress not only limit agriculture all over the world [2,3], but also dramatically compromise the nutritional value of agricultural plants [4]. Therefore, understanding of the mechanisms behind the loss of plant productivity and nutritional value under water stress conditions is important for global food security, especially in respect of such an important source of food protein as legumes [5]. 

In general, plant response to water deficit and accompanying osmotic stress result in drought resistance, which can be achieved via three principal strategies—(i) drought escape, (ii) drought avoidance, and (iii) drought tolerance [6]. Thereby, at the early steps, dehydration triggers stomata closure, which is regulated by abscisic acid (ABA)-dependent signaling pathways [7]. It results in an overload of chloroplast and mitochondrial electron transport chains, and overproduction of reactive oxygen and nitrogen species (ROS and RNS, respectively) [8]. These highly-reactive intermediates, on one hand, are involved in signaling pathways underlying drought tolerance [9], on the other—impact on enhancement of monosaccharide autoxidation and lipid peroxidation, which are known to be accompanied with damage of cellular biopolymers [10].

Due to their ability to form root nodules, i.e., symbiotic association with soil rhizobial bacteria, legumes represent one of the most drought-responsive crop plants. Indeed, as osmotic stress disrupts cellular ionic and osmotic equilibrium [11], nodules are known to be highly sensitive to osmotic stress [12]. Reduced soil water potential (Ψ_w_) also affects diffusion of oxygen via the root epidermis, directly affecting the nodule function [13]. Finally, symbiotic nitrogen fixation in nodules is highly sensitive to drought-induced osmotic misbalance: dehydration negatively affects nitrogen accumulation and might compromise yields of legume crops [14].

**Figure 1:**
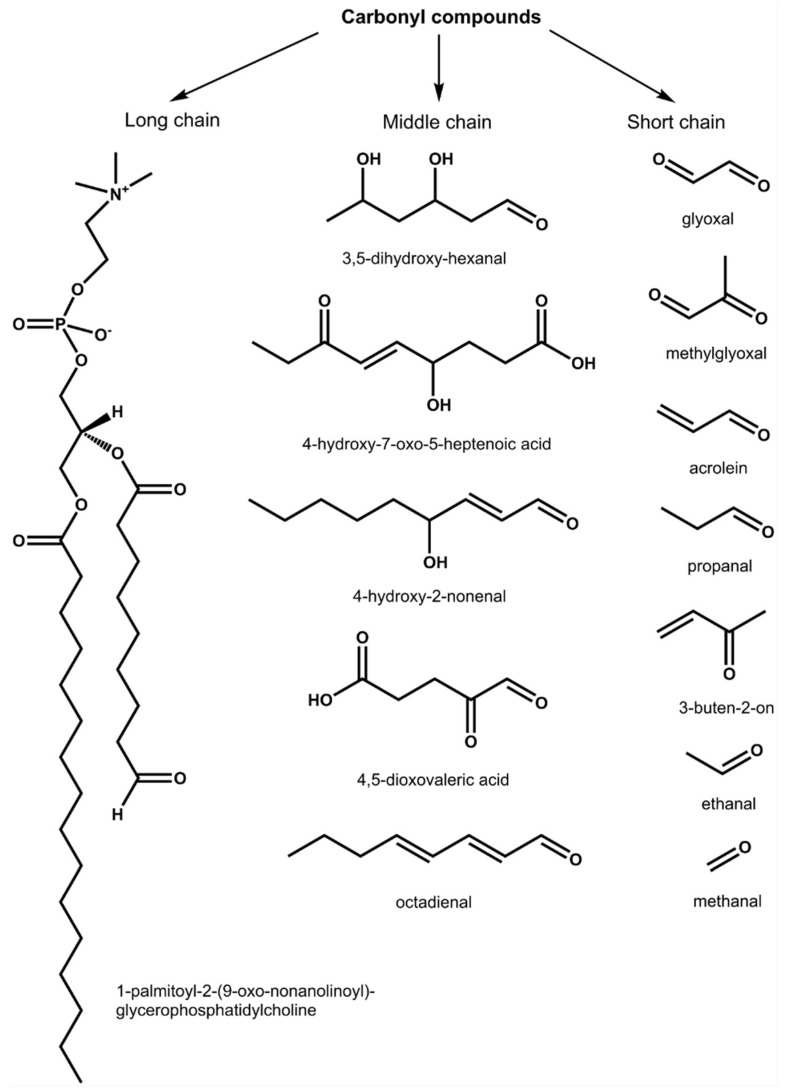
The main structural groups of reactive carbonyl compounds (RCCs) with representative structures, detectable in biological systems as derivatives of 7-(diethylamino)coumarin-3-carbohydrazide (CHH).

The observed changes in root nodule functional activity can be underlied by oxidative stress, which accompanies plant response to dehydration and resulting osmotic stress. As lipids and sugars are the primary targets of cellular ROS and NOS, plant response to dehydration is ultimately accompanied by formation of lipid hydroperoxides. Their subsequent degradation leads to formation of reactive carbonyl compounds (RCCs), represented by a broad array of saturated and unsaturated aldehydes, ketones and oxocarboxylic acids (Figure 1) [15], which readily react with nucleic acids [16] and proteins [17]. The patterns of such modifications can be derived from the knowledge of the carbonyl metabolome [18].

During the last decade RCCs attracted a special attention of plant biologists as prospective regulatory factors and important players of cellular signaling pathways mediating stress response. Thereby, selective modification of specific proteins with RCCs represents early events accompanying stress response [19]. In contrast to ROS, RCCs have typical half-lives from minutes to hours that allows them interacting with targets far from the sites of their generation [20]. The modulating effect of RCCs on gene expression was confirmed by their exogenic application [21]. For example, (*E*)-2-hexenal was shown to activate a wide range of genes involved in protection against pathogen attack in *A. thaliana*, whereas acrolein and methylvinyl ketone triggered upregulation of the pathogenesis-related gene *HEL* [22,23]. Malondialdehyde (MDA) showed a broader effect—its exogenic application triggered expression of multiple genes involved in protection against a broad array of environmental stresses in *A. thaliana* [24]. Interestingly, exogenic application of 12-oxophytodienic acid (OPDA) to *A. thaliana* plants induced a set of defence genes which was distinctly different from those activated by jasmonate and methyl jasmonate [25]. These responses were accompanied with covalent modification of proteins with RCCs. Thus, salt stress was shown to be accompanied with covalent binding of 4-hydroxy-(*E*)-2-nonenal to cytosolic, peroxisomal, chloroplast, mitochondrial and apoplast proteins [21]. During the heat stress in spinach leaves, OEC33 protein in photosystem II was modified with MDA and acrolein, but in *A. thaliana* leaves the antenna LHCII protein was sensitively modified with MDA [26]. Also, such modifications were described for legume nodules, where 238 carbonylated proteins derived from 12 carbonylation pathways were identified [27].

Due to the low stability of RCCs and their high reactivity towards nucleophiles, analysis of these compounds is a challenging task. In the most reliable way, the RCC-metabolome can be addressed by carbonyl-specific derivatization agents, like *o*-phenylenediamine, *O*-alkyl hydroxylamines, hydrazines, cysteine amines, and aminoguanidine or similar carbonyl-trapping compounds [28,29]. However, application of each derivatization agent is typically limited to some specific classes of carbonyl-containing molecules—either α-dicarbonyls, like glyoxal, methylglyoxal and 3-deoxyglucasone, or hydroxyaldehydes and ketones, which can be, in turn, either short or long chained [28,30]. In this context, application of such a derivatization reagent as 7-(diethylamino)coumarin-3-carbohydrazide (CHH) might be advantageous [15]. On one hand, the tertiary amino group, present in the CHH structure, allows superior ionization of derivatives in the positive ion mode. Thereby, the specific loss of the coumarin moiety and characteristic fragmentation patterns of RCC derivatives can be used for unambiguous annotation of the analytes as carbonyls and structure assignment, respectively [31]. On the other hand, this reagent gives access to the simultaneous extraction and detection of both low and high molecular weight RCCs [15]. Therefore, here we apply this compound for a comprehensive characterization of the carbonyl metabolome of pea root nodules (to the best of our knowledge, the first plant object where comprehensive carbonyl metabolome was described so far) and the effects of experimental polyethylene glycol (PEG)-derived osmotic stress on the patterns of carbonyl metabolites. To address the latter aspect in a proper way, we also consider stability of derivatives, representing different RCCs and propose a strategy for correction of the observed stability differences.

## 2. Results

### 2.1. Establishment of the Drought Stress Model: Plant Growth and Harvesting

The planted seeds germinated quantitatively and produced healthy seedlings (Supplementary Materials, Figure S1-1A) with no abnormalities observed during the first week of growing on vermiculite (Figure S1-1B) and later on in the hydroponic system (Figure S1-1C). No visible differences in morphology of individual plants could be observed before stress application. However, the plants subjected to osmotic stress (5–20% *w*/*v* PEG8000) showed clear signs of dehydration—loss of leaf turgor, local leaf regions of chlorosis and necrosis already on the next day after application of the PEG-derived osmotic stress. The highest dosage of PEG8000 (25% *w*/*v*) resulted in systemic necrosis and was not considered in further experiments. Necrosis and other visual damage observed in the treated plants could be associated with PEG toxicity. The control plants did not demonstrate any signs of dehydration and toxicity.

### 2.2. Physiological and Biochemical Characterization of Plant Stress Response

**Figure 2:**
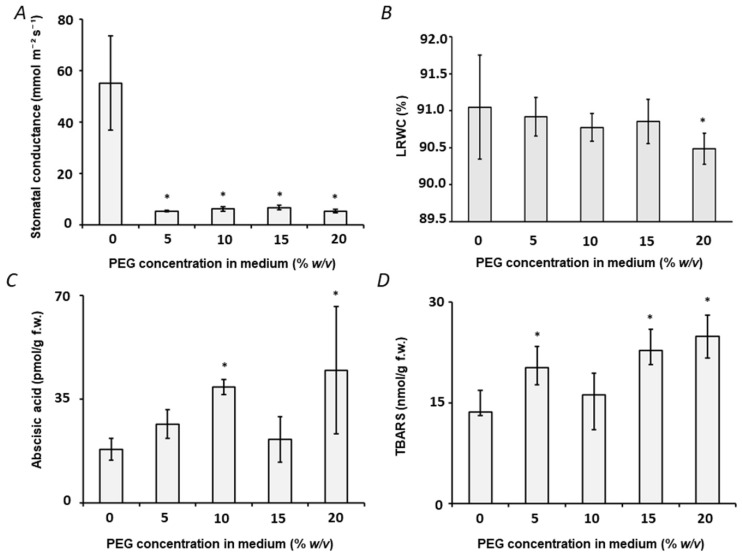
Physiological and biochemical characterization of pea *(P. sativum)* plant response to osmotic stress applied by transfer of two week-old plants to aqueous growth medium (with the composition specified in the Material and method part) supplemented with 5, 10, 15 and 20% (*w*/*v*) PEG 8000 for one week (controls were grown in PEG-free medium). The plant stress response was characterized at the end of the treatment period (seven days) with stomatal conductance (**A**), leaf relative water content (LRWC, (**B**)), content of abscisic acid (ABA, (**C**)) and TBARS—thiobarbituric acid reactive substances (MDA equivalents, (**D**)). Statistically significant differences (two-side Student *t*-test for two groups with the same variance, *p* ≤ 0.05) in comparison to the plants grown in absence of PEG are marked with asterisk.

The severity of osmotic stress was estimated by intensity of plant stress response. For this, physiological and biochemical stress markers were assessed under the osmotic stress conditions of increasing severity. For this, 5, 10, 15 and 20% (*w*/*v*) PEG 8000 were supplemented to the growth medium for seven days, that resulted in calculated water potential (Ψ_w_) values of −0.27, −0.285, −0.3, −0.316, and −0.33 MPa, respectively. After one week of growth under hydroponic conditions, clear drought-related physiological effects could be observed with all adverse osmotic conditions tested. Thus, already the minimal PEG concentration resulted in a significant reduction of stomatal conductance (90.3 mmol m^−2^ s^−1^, *t*-test *p* ≤ 0.05) (Figure 2A, Supplementary Materials Information 2, Table S2-1). However, only the highest Ψ_w_ value resulted in significant dehydration (Figure 2B and Table S2-2), which was, however, less pronounced in comparison to the water loss associated with osmotic stress in *Arabidopsis* [32]. Of course, it cannot be excluded that the observed changes are related to the toxic effects associated with application of PEG [8]. On the other hand, the toxicity of high-molecular-weight PEG is typically associated with direct contact of liquid PEG-containing medium with damaged roots. Such damage allows entry of PEG in the vascular system that results in its physical blockage. Therefore, wounding and damage of roots were avoided during the experiments. Interestingly, although no effect on root length could be observed (Figure S1-2A and Table S2-3), low PEG concentrations (5% *w*/*v*) resulted in increase of leaf chlorophyll contents (Figure S1-2B and Table S2-4). 

Analysis of biochemical stress markers revealed up to two-fold increase of abscisic acid leaf contents after PEG treatment (*t*-test *p* ≤ 0.05, Figure 2C, Table S2-5), whereas changes in the contents of jasmonic acid (JA), its precursor OPDA and active form jasmonyl isoleucine conjugate (JA-Ile) were not significant (Figure S1-2C and Table S2-6). The unexpected dynamics of the phytohormone levels might be the illustration of successful activation of adaptive mechanisms and completence of metabolic adjustment. This response can be heterogenous within the plant group that also might be the reason for the absence of significant differences at the dosage of 10% *w*/*v* PEG. Not less importantly, even the minimal stress dosage (Ψ_w_ = −0.27) resulted in enhanced ROS production and development of oxidative stress, as it can be judged from a significant 5–24% increase of lipid peroxidation (assessed as malondialdehyde, MDA, production, Figure 2D and Table S2-7). Surprisingly, no significant changes in the contents of TBARS were observed after treatment with 10% (*w*/*v*) PEG8000. This might be attributed to heterogenicity in stress adjustment patterns, which cannot be excluded when small groups are addressed. On the other hand, no effect on ascorbic acid biosynthesis could be observed (Figure S1-2D and Table S2-8). As the over goal of this study was to characterize the alterations in the profiles of reactive carbonyl compounds (RCCs), here we aimed moderate severity of the stress response, which is known to trigger oxidative degradation of lipids (manifested as up-regulation of TBARS) in legumes [33]. As the symptoms of moderate stress, we treat 90% reduction of stomatal conductance and essential increase of ABA contents, as the principal markers of osmotic stress [34] in combination with increase in tissue contents of TBARS, which is the critical parameter in terms of understanding ROS-mediated lipid damage [35].

**Figure 3:**
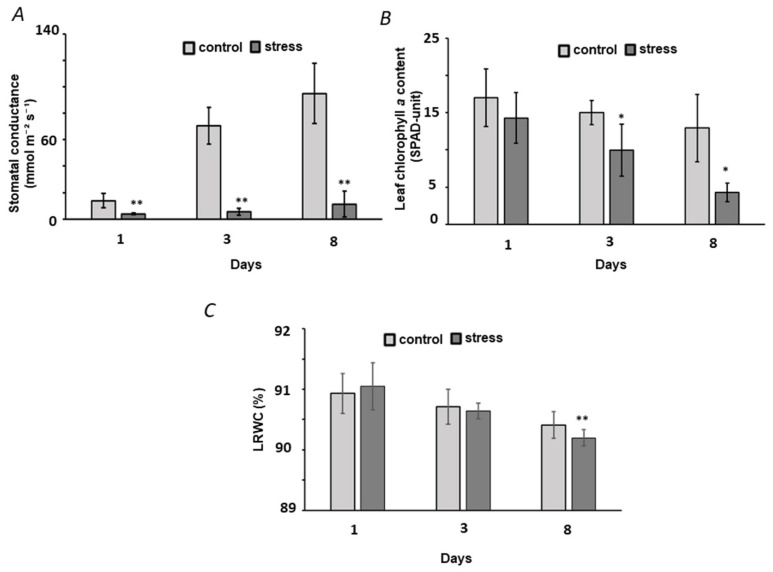
Physiological characterization of pea (*P. sativum*) plant response to osmotic stress applied by transfer of two week-old plants to aqueous growth medium (composition is specified in the Material and method part) supplemented with 10% (*w*/*v*) PEG 8000 for 1, 3 and 8 days. Controls were grown in PEG-free medium and harvested at the same times. The plant stress response was characterized with stomatal conductance (**A**), chlorophyll *a* contents (**B**), and LRWC (**C**). Statistically significant differences (two-side Student *t*-test for two groups with the same variance, * *p* ≤ 0.05, ** *p* ≤ 0.01) in comparison to the plants grown in absence of PEG are marked with asterisk.

Based on these considerations, our previous research [12] and other published data, which indicated 10% (*w*/*v*) PEG as the physiologically relevant stress-inducing dosage [36,37], we selected this PEG concentration for further experiments. To address the dynamics of the stress response we characterized dynamics of the plant physiological parameters to the selected conditions (10% *w*/*v* PEG 8000) over the whole time of the stress application. The results indicated a pronounced alteration in stomatal conductance over eight days of the experiment: this parameter demonstrated a nine-fold decrease on the third day of treatment, whereas five days later this difference was twelve-fold (Figure 3A and Table S2-9). Approximately a three-fold decrease in chlorophyll *a* content (Figure 3B and Table S2-10) and a significant drop in leaf relative water content (LRWC) (Figure 3C and Table S2-11) could be observed after eight days of treatment. These data confirmed one week upon stressor application as an appropriate sampling time point with clearly manifested stress response.

### 2.3. Quantification of Prospective CHH Derivatives of RCCs by UHPLC-ESI-LIT-Orbitrap-MS

Due to the high resolution (*m*/Δ*m*_200_ = 30000) and mass accuracy (below 5 ppm) specified for the measurements with the Orbitrap Elite instrument, derivatized RCCs could be reliably annotated in pooled preparations (obtained by combination of all individual samples) by their t_R_ and *m*/*z*. Specifically, for all *m*/*z* values below 3 ppm, elemental composition could be calculated and verified for agreement with the presence of the (diethylamino)coumarin moiety in corresponding structures. For some analytes possible structures could be proposed (i.e., tentatively assigned) based on the derived elemental composition and earlier published data [15,31], whereas the others were treated as unknowns. For all features satisfying these two requirements (and treated, therefore, as prospective CHH derivatives of RCCs) characteristic extracted ion chromatograms (XICs) (*m*/*z* ± 0.02) were built for the quality control pooled samples. This strategy revealed in total 194 RCC derivatives demonstrating signal intensity in extracted ion chromatograms above 1.0 × 10^5^ counts (Supplementary Materials Information 3, Table S3-1).

**Figure 4:**
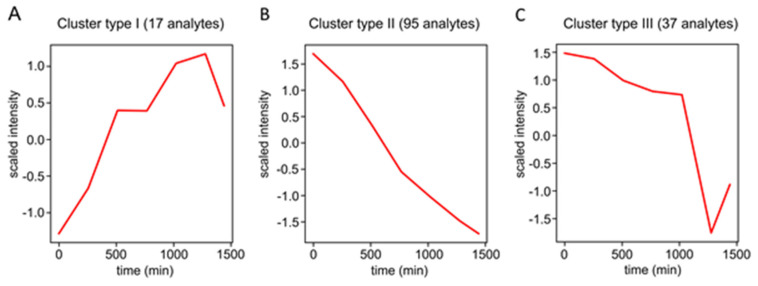
Types of stability profiles/degradation kinetics observed for CHH derivatives of RCCs and characterized by continuous intensity increase (**A**), intensity decrease (**B**) and slow intensity decrease followed with a rapid drop afterwards (**C**). The centroids of the final clusters are defined as the average pattern from time curves of individual CHH derivatives of RCCs in a certain cluster produced using K-means clustering.

At the next step, the relative abundances of these 194 prospective RCC derivatives in PEG-treated and control root nodules were addressed by the label free quantification approach. For this, individual features were integrated in the Quan tool of the Xcalibur software. Thereby, a special attention was paid on stability of the derivatives. Indeed, as was earlier reported by Milic et al., CHH derivatives are characterized with compromised stability during their storage in frozen state [31], although their short-term stability in a refrigerator or autosampler (4–8 °C) still remains unknown. Therefore, here we addressed this issue in more detail. Thus, to probe the stability of CHH derivatives of RCCs and to characterize their degradation kinetics, we quantified peak areas of all 194 prospective RCC derivatives across the set of quality controls (pools obtained by combining of all individual samples and injected throughout the sample batch each 255 min) and plotted degradation kinetics of each individual RCC compound (Figure S1-3) during 24 h, that covers the measurement duration of a standard metabolomics experiment. Afterwards, the clustering analysis was accomplished. Based on its results, the degradation kinetics curves for each individual RCC compound could be plotted, and the groups of RCCs demonstrating similar behavior could be visualized (Figure S1-3). For all groups of the kinetics curves (clusters) obtained by our workflow (described in the Section 4 and in more detail in Protocol S1-1) corresponding centroids were obtained. These centroids could be considered as a graphical representation of kinetics behavior derived for the corresponding sets of individual prospective CHH-derivatives of RCCs (Figure 4).

The analysis revealed essential differences in the time curves of individual prospective RCC derivatives, which could, however, be grouped into a limited number of generalized types of kinetics profiles. Thus, the relative abundance of RCC derivatives could continuously increase in a linear manner (kinetics type I, 17 RCC derivatives), continuously linearly decrease (kinetics type II, 95 analytes) or slightly and gradually decrease within the first 1000 min of observation with a rapid drop afterwards (kinetics type III, 37 compounds). These three clusters were featured with different degrees of abundance changes of individual analytes within the observed time frame (Figure 4).

Thus, cluster I comprised mostly (14 of 17) compounds, featured with relative standard deviations (RSDs %) within 10% over the observation time, which can be considered as low. In contrast, only approximately 40% of the Cluster II (39 of 95 analytes) demonstrated this behavior, while the remaining 56 features had RSDs ranging from 10 to 85%. The cluster III was mostly (21 analytes, 57%) represented by relatively stable compounds (RSD within 10%), whereas the rest (16 analytes) demonstrated RSDs up to 61%. Finally, 45 RCCs showed no essential alterations in relative contents within the analysis time, i.e., demonstrated a steady state behavior with the most of the features showing RSDs within 10% and not exceeding 17% (kinetics types IV and V, Figure S1-4).

We proposed that these different types of stability profiles could be attributed to specific structural features of RCCs. To check this hypothesis, we analyzed elemental composition of all features and interpreted tandem mass spectra of the top-five candidates representing each cluster. However, no cluster-related bias in elemental composition of derivatives was observed, i.e., cluster analysis did not interfere with the number of oxygen atoms in the molecule and with the chain length. Moreover, analysis of only top-five MS/MS spectra was sufficient to reject our hypothesis, i.e., to demonstrate that clustering of CHH-derivatives by their stability kinetics profiles was not related to chemical classes of the analyzed RCCs (data not shown).

**Figure 5:**
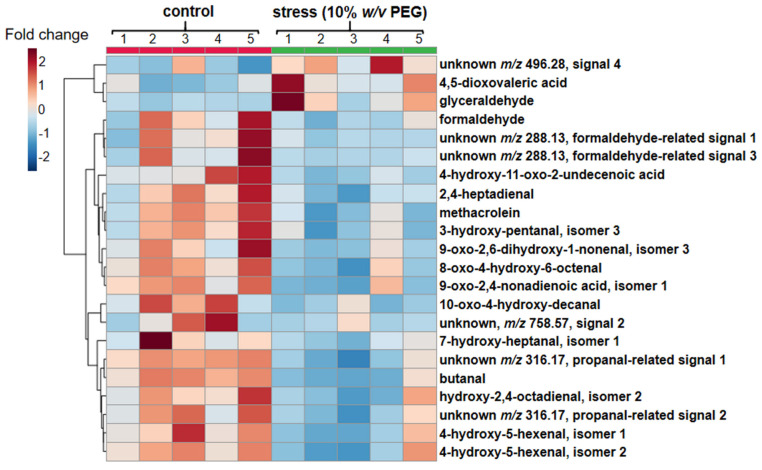
Hierarchical clustering analysis and heatmap representation illustrating stress-dependent differential regulation of 22 RCCs in the root nodules of control and experimental pea (*P. sativum*) plants, treated with 10% (*w*/*v*) PEG8000 supplemented to the hydroponic growth medium for one week.

Nevertheless, despite the essential intra-cluster structural variability, the compounds within each cluster could be subjected to regression analysis and the obtained results could be further applied to the experimental set of samples (randomized across the whole sample set) for intensity normalization and correction of the differences for the magnitude of analyte abundance alterations within the analysis time. The comparison of the PEG-treated and control groups by the Student’s *t*-test revealed 22 differentially abundant analytes (Figure 5). Among them, 19 prospective RCCs were downregulated in the root nodules of the pea plants treated by osmotic stress. The magnitude of these alterations ranged from 1.5 to 5.3 fold (formaldehyde-related signals of unknowns at t_R_ 6.6 and 9.9 min, respectively, Table 1). On the other hand, three analytes were significantly upregulated in the PEG-treated nodules: 4,5-dioxovaleric acid (2.1-fold), glyceraldehyde (2.7-fold) and one unknown carbonyl metabolite (1.7-fold, Table 1).

As can be shown from Figure S1-5, the results of the statistical analysis were essentially different in the absence of the correction for derivative stability. Thus, only one of three up-regulated species could be detected as such without correction (thereby, no differences in fold change were observed), whereas the other two were not recognized as differentially abundant anymore. Among the 19 down-regulated species 18 matched between the corrected and non-corrected datasets, whereas only one compound, namely pentanal, was present only in the non-corrected dataset. Thus, the computational correction for analyte stability was strongly mandatory to obtain an adequate result.

For cross-validation of the obtained result, we addressed the contents of 4,5-dioxovaleric acid and glyceraldehyde in one of the earlier time points—on the third day after stress application. Both analytes were significantly (*p* ≤ 0.05) up-regulated in the root nodules of the stressed plants in comparison to the time-matched untreated controls (Figure S1-6)—1.6- and 1.4-fold, respectively. This result was in a good agreement with our concept and the values obtained four days later (2.1- and 2.7-fold, respectively).

### 2.4. Structure Characterization of Stress-Regulated Nodule RCCs by MS/MS

**Figure 6:**
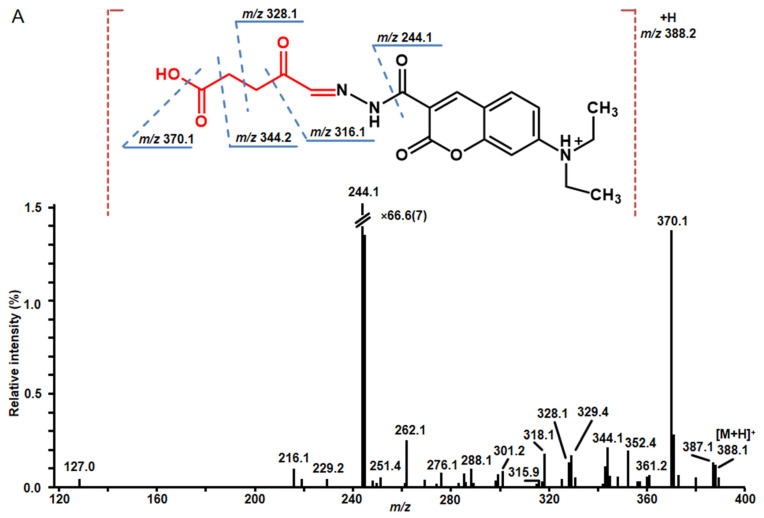

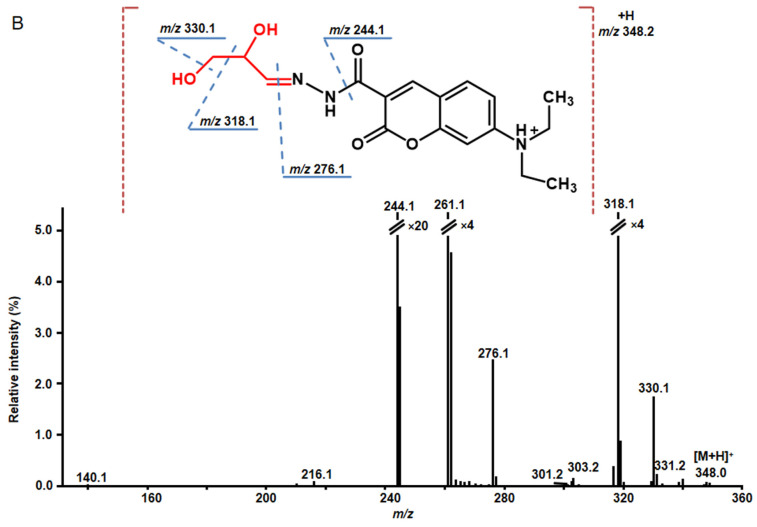
Fragmentation spectra of the CHH derivatives of 4,5-dioxovaleric acid (**A**) (*m*/*z* 388.1503) and glyceraldehyde (**B**) (*m*/*z* 348.1554) differentially regulated in the root nodules of control and experimental pea (*P. sativum*) plants, treated with 10% (*w*/*v*) PEG 8000 supplemented to the hydroponic growth medium for one week. The MS/MS spectra were acquired in positive ion mode using collision-induced dissociation (CID) functionality of the linear ion trap (LIT) mass analyzer (35% (**A**) and 30% (**B**) normalized collision energy) of the Orbitrap Elite mass spectrometer. The part of the derivative corresponding to the RCC structure is marked red.

The identity of significantly (*t*-test, *p* ≤ 0.05) up- and down-regulated RCCs was confirmed by tandem mass spectrometry. For 21 compounds MS/MS spectra were successfully acquired in comprehensive data-dependent acquisition and targeted MS/MS experiments (Table 1). The structures verified by manual interpretation of MS/MS spectra as exemplified for 4,5-dioxovaleric acid and glyceraldehyde in Figure 6 and presented in more detail in Figure S1-7.

All CHH derivatives demonstrated rich tandem mass spectrometric patterns, informative in terms of deriving the structures of corresponding RCCs. Thus, fragmentation patterns of all CHH-derivatives dominated with characteristic fragments at *m*/*z* 244 and *m*/*z* 262, corresponding to the neutral losses of RCC and additionally hydrazine moiety (-N_2_H_4_, -32 u), and amino group (-NH_2_, -16 u). These signals can be considered as diagnostic for RCC derivatives. Further fragmentation of CHH-RCCs derivatives was represented by the signals corresponding to fragmentation of hydrocarbon backbone. Collision-induced dissociation tandem mass spectra of alkanals do not contain any aldehyde specific ions, but in alkenals fragmentation next to the double bond is present. Fragmentation of hydroxy-alkans and –alkens is characterized by the presence of relatively intense peaks corresponding to the ions formed by the cleavage of the hydroxyl group (Figure 6B). Oxo-carboxylic acids showed rich tandem mass spectra containing abundant neutral losses of water, formic acid (-CH_2_O_2_, -46 u) or CO_2_ (-44 u) (Figure 6A). Unfortunately, due to difficulties in identification of isomers, the structure of lipid-derived RCC at *m*/*z* 758.5685 could not be unambiguously assigned (Figure S1-8)

## 3. Discussion

### 3.1. Establishment of Osmotic Stress in Pea plants

As was comprehensively summarized recently by Osmolovskaya et al. [6], to date, drought can be efficiently modeled by multiple approaches based on soil, agar and liquid mediums. Recently, we demonstrated, that application of polyethylene glycol (PEG) 8000 g in the *Arabidopsis* agar-based PEG infusion model yields physiological and metabolic alterations, which were closely similar to those induced by physiological drought [8]. This fact indicated that PEG-induced osmotic stress can be considered as an adequate model of drought, although its results require further confirmation in soil-based setups, which are obviously more physiologically relevant. Therefore, here we make the first step and setup the PEG-based model of osmotic stress with the intention to extend it to drought. In contrast to the agar-based model, supplementation of PEG (typically 6000 or 8000) provides the most direct and precise way to define the water potential of the growth medium at the desired level. Indeed, this setup of modeling drought by osmotic stress can be applied not only to seeds and seedlings, but also to developed plants at any later stage of ontogenesis [38]. In our previous work we confirmed applicability of this approach [39]. Moreover, recently, we applied the same PEG-based aqueous model to the study of the alterations in pea nodule proteome induced by osmotic stress [12]. Interestingly, the resulted stress-related patterns of differential protein expression were in good agreement with the previously published data of Gonzalez et al. [40], although the authors used a much more sophisticated and physiologically straightforward split-root approach. Based on this fact and keeping in mind our earlier osmotic stress experiments with mature pea plants at the stage of seed filling [39], we decided for this model as the most suitable for the analysis of carbonyl metabolome dynamics.

Based on the overall goal of our study (which was probing the nodule patterns of RCCs and their changes under drought conditions) and its setup, we selected the panel of stress markers for adequate characterization of dehydration-related stress induced by application of PEG. Based on this, a PEG dosage corresponding to moderate stress (i.e., agriculturally relevant situation) could be selected. Thereby, the selection of the markers was supposed to allow: (i) confirmation of stress, (ii) confirmation of the oxidative stress conditions which underlie lipid peroxidation and formation of RCCs and (iii) sampling low amounts of plant tissue, as hydroponically grown three-week old plants have relatively small leaves. In the context of these considerations, we decided for a broad panel of physiological parameters—LRWC, stomatal conductance, activity of photosystem II, chlorophyll content, which were accompanied with assessment of the biochemical markers for reactive oxygen species (ROS), antioxidants and low-molecular weight regulators (hormones). Thereby, we selected measurement of thiobarbituric acid-reactive substances (TBARS), rather than H_2_O_2_ and antioxidant enzymes, as the marker of the tissue oxidative status. Indeed, H_2_O_2_ and H_2_O_2_-degrading enzymes are the key players of ROS signaling [41], and they are characteristic for early steps of the stress response, i.e., for minimal stress, stimulating acclimation and adaptation to environmental clues [42]. On the other hand, H_2_O_2_ only minimally impacts on formation of RCCs, whereas the hydroxyl radical (OH^.^) is the principal contributor [43], and analysis of TBARS is the most relevant ROS marker in the context of lipid peroxidation [44] and RCC formation [45]. We are keeping in mind also, that closure of stomata and simultaneous increase in ABA contents are the main markers of drought [34], basically sufficient for the conformation of osmotic stress. Importantly, besides the above-mentioned considerations, determination of H_2_O_2_ would require much material (at least 100 mg × 3 replicates according the method established in our lab), although its necessity seems to be questionable as, from the physiological point of view, ABA-dependent stomata closure is underlied by H_2_O_2_ [46]. One need consider also the fact that antioxidant enzymes were shown to be inefficient in the PEG-based model of osmotic stress in pea [37].

Based on the comprehensive literature mining and assuming (*i*) large inter-cultivar variability known for pea and (*ii*) higher stress tolerance of considered here juvenile plants, we probed five PEG8000 concentrations from 5 to 25% (*w*/*v*). Predictably, as can be observed from the physiological data, already the minimal PEG concentration induced a pronounced stress response. This fact could be clearly illustrated by a non-saturating minimal decrease of stomatal conductance (Figure 2A), slight adaptive increase of chlorophyll contents (Figure S1-2B), increase of abscisic acid contents and up-regulation of TBARS (expressed as MDA-equivalents, Figure 2C,D). This response pattern was already observed in our previous work, done in the same stress model [12]. Most probably, these events can be attributed to the effects related to H_2_O_2_ as the main ROS-related stress regulator and to antioxidant enzymes which finely tune H_2_O_2_–related signaling. As these effects are behind the scope of this work, the minimal stressor concentration was not selected for further experiments.

On the other hand, 10% (*w*/*v*) PEG yielded an osmotic stress response, to our opinion, physiologically corresponding to moderate drought. We came to this conclusion, as drop in stomatal conductance became saturating at this stress dosage in parallel to the progressing accumulation of abscisic acid (Figure 2). This was in agreement with the fact that LRWC, chlorophyll, total ascorbate and MDA contents recovered to the control levels after the plant growth at this stress dosage during seven days. The decrease in MDA levels at 10% (*w*/*v*) in comparison to the lowest (5% *w*/*v*) PEG concentration was already shown before [12]. Obviously, this indicated the success of metabolic adjustment and stress adaptation in the conditions when the osmotic capacity of cells is not reached. The higher PEG dosages resulted in severe stress, which was accompanied with saturation of tissue antioxidant capacity at the background of high MDA, ascorbate and jasmonate levels accompanied with full activation of antioxidant enzymes. Such a physiological state was behind the scope of this work as absolutely irrelevant from the points of both biology and agriculture due to complete loss of rhizobial symbiosis at these conditions. Remarkably, 10% (*w*/*v*) is quite commonly used for probing drought protectors and growth regulators in pea [36,37] and was also successfully employed in our previous studies [12]. This additionally supported our decision to perform all further experiments with this concentration.

The observed here patterns of stress markers corresponded well to the results of the comprehensive study of Basal et al. reporting the physiological effects of different PEG concentrations, applied at different stages of legume (specifically, soybean) ontogenesis [38]. Indeed, the continuous and saturating decrease in stomatal conductance with increase of PEG percentage, as well as adaptive slight up-regulation of chlorophyll contents at low PEG concentrations were observed in our study as well (Figure 2A and Figure S1-2B). The phytohormone response was in good agreement with our earlier data, acquired in the experiments with *Arabidopsis* [8] and pea [39] stress models.

Predictably, as can be seen from Figure 3, the severity of osmotic stress gradually increased with time. Thereby, the observed here dynamics of stomatal conductance (Figure 3A) generally reproduced the curves reported for terminate drought in chickpea [47], that indicated physiological relevance of our model. Only late and minimal drop in chlorophyll contents (appearing to be significant on the eighth day of the experiment—Figure 3B) was in agreement with the moderate character of the observed stress. As can be seen from Figure 3C, only minimal decrease of LRWC in PEG-treated plants in comparison to the non-treated controls could be visible after eight days. This might indicate just a minimal degree of dehydration that was also in agreement with the applied here moderate stress severity.

Thus, to summarize, our stress model appears to be physiologically relevant and seems to be in a good agreement with: (i) earlier data on pea obtained with both PEG- and soil/stop watering-based models, (ii) the PEG data acquired with Arabidopsis and other model plants and (iii) the data obtained by orthogonal techniques—e.g., proteomics, which appeared to be compatible between the models.

### 3.2. Analysis of RCCs

In general, analysis of RCCs is challenging [28], and relies, therefore, mostly on targeted strategies, i.e., quantification of selected (and, typically, small-sized) sets of RCCs with well-defined properties by stable isotope dilution [48] and label-free techniques [49]. In contrast, only few studies describe comprehensive quantitative profiling of RCCs [50], none of which, however, dealing with plants. Unfortunately, the targeted approaches (employed so far in plant RCC analytics) deliver only a small part of the information on the whole carbonyl metabolome, typically, not exceeding several dozens of analytes [51]. This is, however, at least an order of magnitude less than presented here. Thus, our study can be considered as the first comprehensive carbonyl profiling survey, accomplished with plants. Moreover, it is the first report with analysis of multiple RCCs in legume root nodules. As these morphological structures house unique symbiotic association—legume-rhizobial symbiosis, the obtained here data need to be treated with care, when interpreted in the context of published leaf RCC datasets.

It is important to note, that in this study we assessed the derivative stability and corrected the acquired data for kinetics profiles of individual RCC-CHH adducts. The importance of this procedure is clearly illustrated with the qualitative and quantitative differences in the RCC abundance patterns obtained after processing of corrected and not corrected data (Figure 5 and Figure S1-5, respectively). The signal drift correction methods based on quality controls are well-known in metabolomics [52], including those that rely on regression analysis [53]. Here we extended this approach to a derivatization-based method relying on a new reagent (CHH), which was not studied in this respect so far. The obtained kinetics profiles can be explained by faster evaporation of solvent in comparison to analyte (cluster I), by derivative degradation (cluster II) and by sequential manifestation of these two factors (cluster III). Although the clusters IV and V were distinguished by the data analysis, both these groups comprise analytes, which did not change their abundance within the experiment time frame. This conclusion is based on relatively low RSD% values (typically not exceeding 10%) for most of the analytes representing these two clusters.

Having in mind a unique metabolic background of legume-rhizobial symbiosis, it was not totally surprising, that the data acquired here for pea root nodules were in conflict with the results of earlier studies on stress-related RCC dynamics in leaf. Indeed, the leaf response to osmotic stress and dehydration is typically manifested with up-regulation of individual RCCs, for example, in the context of ABA-related reactions [32,54]. Therefore, based on the results of literature mining, when planning our experiments, we hypothesized that drought treatment would result in strong up-regulation of nodule RCCs, as it happens in plant shoots. On one hand, we expected a stress-related increase in the levels of oxidative stress in nodules [55] that might be accompanied with enhanced peroxidation of membrane lipids and oxidation of nodule proteins. On the other hand, a dramatic drought-related suppression of the nodule antioxidant defense and resulting decrease of their antioxidant capacity are well-documented (that can be exemplified by a classical work of the Becana’s group [56]) and could be expected here as well.

### 3.3. Stress-Related Dynamics of Nodule RCCs

In contrast to our expectations, the nodule response to PEG-related osmotic stress differed essentially from the scenario described for the leaf and was accompanied with down-regulation of multiple RCCs (Figure 5). Despite this discrepancy with the available data on RCC patterns in leaf [54], this result was in a good agreement with down-glycation of nodule proteins, recently demonstrated in the same PEG-based model [12]. As protein glycation is tightly associated with carbonyl stress (i.e., formation of advance glycation end products is mostly mediated by α-dicarbonyls) [10], the observed here stress-related down-regulation of RCCs might be underlied by the same mechanisms.

As can be seen in Figure 5, the down-regulated species are represented mostly with short-chained RCCs, at least some of which are known to modify cellular biopolymers [57]. As derivatization with CHH gives a reliable access to long-chained RCCs as well [15], it can be assumed that this group of reactive carbonyls just minimally affected by experimental osmotic stress (as only one representative of long-chained RCCs was tentatively identified). However, this assumption requires additional check with in vitro trials and MS^n^ experiments. Indeed, the annotation of short-chained metabolites can be also a result of in-source fragmentation: for example, appearance of the formaldehyde-related artifacts at three different retention times (3, 4 and 17 in Table 1) supports this consideration.

One of the mechanisms behind suppression of short-chained RCCs might employ stronger (in comparison to leaf) stress-related up-regulation of the enzymes, involved in protection against carbonyl stress in plants. Such systems are well-described for photosynthetically active plant organs and typically include: (i) aldehyde dehydrogenase, oxidizing aldehydes to carboxylic acids in a nicotinamide adenine dinucleotide (NAD^+^)-dependent manner, (ii) aldehyde reductase to reduce aldehydes or ketones to corresponding alcohols with nicotinamide adenine dinucleotide phosphate (NAD(P)H), and (iii) 2-alkenal reductase and alkenal/one oxidoreductase to reduce RCCs at the carbonyl-conjugated C-C double bond with NAD(P)H. Also several aldehydes, including acrolein, can be scavenged by glutathione-S-transferases [58]. During the last decades, all these enzymes were confirmed as important contributors of redox homeostasis in legume root nodules. Thus, already long time ago, Suganuma and Yamamoto showed that aldehyde dehydrogenase is activated in nodule symbiosis in comparison to free living bacteria [59]. Moreover, aldehyde reductase was shown to be upregulated in legume roots in response to moderate/short-termed drought [60], whereas several aldo-keto reductases were shown to contribute in drought stress defense in *Medicago truncatula* [61]. Finally, several glutathione-S-transferases were shown as important players of the stress protection system in soybean root nodules [62].

On the other hand, RCCs might be activity enhancers of ROS-scavenging enzymes, as it was demonstrated for *A. thaliana* leaf catalase and ascorbate peroxidase upon external application of acrolein, 4-hydroxy-hexenal, and 4-hydroxy-2-nonenal [63]. According to Cao et al., this effect can be mediated by the early NO burst, which might be triggered by NOS-like enzymes in root in response to osmotic stress [64]. Involvement of these and other redox-related post-translational modifications in stress response of legume root nodules is being intensively studied [65].

Another explanation of the observed dynamics of the reactive carbonyls would employ suppression of RCC production via antioxidant (more precisely—ROS scavenging) activity of phenolic metabolites, which are (i) known to be abundant in pea root nodules (especially phenolic acids which serve as signaling molecules in legume-rhizobial symbiosis) [66] and (ii) are known as efficient RCC scavengers (carbonyl traps). Indeed, the RCC-trapping properties of flavan-3-ols [67], as well as condensed [68] and hydrolysable [69] tannins, which are able to protect proteins from carbonyl-related modifications are well-known.

Due to high reactivity of amino groups towards α-dicarbonyls [70], the role of RCC traps can be efficiently accomplished by free amino acids and polyamines as well. In general, taking into account the mechanism of nitrogen fixation, relying on metabolism of arginine [71] and the fact of amino acid accumulation under drought conditions in legumes [72], involvement of free amino acids in carbonyl trapping seems to be rather likely. To address this assumption, we considered the changes in primary metabolite profiles associated with osmotic stress. The metabolomics experiments revealed up-regulation of several amino acids in the drought-treated group in comparison to controls: L-leucine (1.3-fold), L-homoserine (2.3-fold), L-aspartic acid (1.7-fold), L-phenylalanine (1.4-fold), L-lysine (3.8-fold), L-tyrosine (3.7-fold) and L-tryptophan (4.4-fold). Although some polyamines were affected by osmotic stress as well (e.g., 2.3-fold upregulation of putrescine), no accumulation of proline was observed, as it was reported for the root nodules of *Medicago sativa* in response to drought [73]. Thus, trapping of RCCs by amino acids, overproduced in terms of osmoregulation, nitrogen storage or energy metabolism [74] appears possible. To verify this hypothesis, analysis of corresponding advanced glycation end products (AGEs) [70] needs to be done and ideally also transferred to a soil-based drought model.

Surprisingly, only three RCCs were upregulated under osmotic stress conditions in nodules. Thus, the contents of 4,5-dioxovaleric acid (DOVA) in stressed plants were higher in comparison to the control group. DOVA is formed via metal-catalyzed oxidation of δ-aminolevulinic acid (ALA) [75]. In mammals DOVA is a marker of oxidative stress, which readily reacts with proteins yielding intra- and inter-molecular cross-links and forms cyclic adducts of adenine and guanine in DNA [76,77]. However, these reactions still need to be confirmed in plants. Remarkably, it was shown that DOVA acts as a strong inhibitor of ALA dehydratase, and its upregulation is accompanied with suppression of porphyrin biosynthesis via inhibition of porphobilinogen formation [78].

In agreement with this, it was shown that drought is accompanied with a pronounced decrease in root nodule leghemoglobin contents [79]. It is well-known that ALA acts as a key intermediate in the early phase of the porphyrin biosynthesis—the principal pathway involved in formation of core heme structure of animal proteins (hemoglobin and myoglobin) and leghemoglobin of the legume root nodules [80]. Thus, independently from the exact pathway of ALA formation—via ALA synthase activity of the bacterial symbiont or via the glutamate-dependent route of the plant host [81], its further incorporation in heme structure is blocked by DOVA. Thus, oxidation of ALA to DOVA might be a contributor in suppression of nitrogen fixation in legume nodules under drought conditions. However, further experiments (also accomplished in soil-based models) are necessary to assess the exact impact of this mechanism in drought-related loss in productivity of legume crops.

Another stress-dependently up-regulated nodule RCC was glyceraldehyde. In nodules it is synthesized in the form of glyceraldehyde-3-phosphate (GAP)—a glycolytic intermediate, which is readily involved in the glyceraldehyde-3-phosphate dehydrogenase (GAPDH) reaction to yield *D*-glycerate-1,3-diphosphate [82]. GAPDH is strongly up-regulated in nitrogen fixing nodules in comparison to roots and represents one of the major routes of carbon supply for bacteroides [59]. Moreover, this strong up-regulation of GAPDH can be explained by high reactivity of GAP, which needs to be promptly detoxified [83].

Water stress ultimately results in suppression of the GAPDH activity and accumulation of GAP in cells. The major mechanism of GAPDH inhibition relies on reversible oxidation of Cys150 catalytic residue with hydrogen peroxide, which yields sulfenic acid [84]. Alternatively, reversible inactivation of GAPDH might rely on S-glutathionylation which results in reversible inhibition of glycolysis and activation of the pentose phosphate pathway (and related NADPH and glutathione synthesis) in response to oxidative stress [85]. Thus, accumulation of GAP (with its sub-sequent non-enzymatic dephosphorylation) might be underlied by a dramatic drop of nitrogen fixation activity under drought conditions and, hence, decrease in the consumption of organics by bacteroides. This chemistry is well-known in mammals where it represents one of the main pathways of *in vivo* methylglyoxal generation [86]. However, alternative pathways of glyceraldehyde formation, e.g., oxidative degradation of ribose [87] need to be considered as well.

To summarize, both regulatory mechanisms (inhibition of ALA dehydratase and GAPDH) rely on oxidative modifications of metabolites or enzymes by ROS, overproduced in presence of PEG-induced osmotic stress. Thus, DOVA and glyceraldehyde might be regulatory metabolites involved in stress-related modulation of nitrogen fixation and antioxidant defense in root nodules that needs now to be addressed in soil-based drought model with complete characterization of stress response in nodules. Further downstream regulatory events are still to be characterized. On one hand, these effects can be linked to lipid signaling via phospholipase D activation by oxidized GAPDH [88] or via modification of cellular proteins [51]. Thus, similarly to recently described involvement of RCCs in regulation of stomatal conductance [89], reactive carbonyls might be regulators of legume-rhizobial symbiosis.

## 4. Materials and Methods

### 4.1. Reagents, Plant Material and Rhizobial Culture

Unless stated otherwise, materials were obtained from the following manufacturers: Carl Roth GmbH & Co. (Karlsruhe, Germany): sodium phosphate dibasic dehydrate (p.a.), polyethylene glycol (PEG) 8000 (p.a.); Honeywell Riedel-de Haen (Seelze, Germany): acetonitrile (LC-MS grade), methanol (LC-MS grade). All other chemicals were purchased from Merck KGaA (Darmstadt, Germany). Water was purified in house on a water conditioning and purification system Millipore Milli-Q Gradient A10 system (resistance 18 mΩ/cm, Merck Millipore, Darmstadt, Germany). Pea (*P. sativum*) seeds of the cultivar SGE and rhizobial culture (*Rizobium leguminosarum* bv. viciae CIAM 1026) were provided by the All-Russia Research Institute of Agricultural Microbiology (St. Petersburg, Russia).

### 4.2. Plant Experiments and the Model of Osmotic Stress

Pea (*Pisum sativum* L., cultivar SGE) seeds were surface sterilized by a 7-min incubation in 33% (*v*/*v*) ethanol, germinated in dark for two days, transferred to polyethylene pots (1 L, two plants per pot) filled with vermiculite, and the seedlings were inoculated with 150 mL of rhizobia (*Rizobium leguminosarum* bv viciae 1026) suspension (optical density, 0.05), supplemented to each pot. After inoculation, the plants were supplemented with a nutrient medium [90] (250 mL per pot), containing 0.75 mmol/L NH_4_NO_3_. The plants (n = 7) were grown under long day conditions (16 h light and 8 h dark) at 22 °C and 75% humidity. One week later, the plants were transferred to aerated hydroponic system, based on the nutrient solution, containing 0.041 mol/L MgSO_4_·7H_2_O, 0.06 mol/L K_2_HPO_4_, 6.5 mmol/L Ca_3_(PO_4_)_2_, 0.81 mmol/L H_3_BO_3_, 0.25 mmol/L (NH_4_)_2_MoO_4_, 0.05 mmol/L KBr, 0.024 mmol/L CuSO_4_·5H_2_O, 0.03 mmol/L KI, 0.0045 mmol/L Al_2_(SO_4_)_3_·18H_2_O, 0.05 mmol/L NaCl, 0.0124 mmol/L MnSO_4_·5H_2_O, 0.007 mmol/L ZnSO_4_·7H_2_O, 0.013 mmol/L NiSO_4_, 0.27 mmol/L NaFeEDTA (pH 7.0–7.2), and grown for two further weeks under the same light regimen with a change of the medium after seven days. Afterwards, the experimental plants were transferred to the growth medium supplemented with 5, 10, 15, 20, and 25% (*w*/*v*) PEG 8000, whereas the control plants were transferred to PEG-free growth medium. One week later, physiological stress parameters were determined for one of the two plants growing in each pot, whereas the second plant (leaves and root nodules separately) was harvested, frozen in liquid nitrogen and ground in a Mixer Mill MM 400 ball mill with a 20 mm stainless steel ball (Retsch, Haan, Germany) at a vibration frequency of 30 Hz for 1 min. For determination of physiological parameters, each technical replicate represented one leaf. The ground material was stored at −80 °C until further analysis.

### 4.3. Physiological and Biochemical Assays

The assessment of adequate physiological and biochemical stress markers relied on the representative panel of methods, established in our group [32,39]. Stomatal conductance (expressed as a specific alteration in the rate of water evaporation from the leaf surface) was assessed with a portable porometer (AP4 Delta-T Devices Ltd., Cottbus, Germany), the relative chlorophyll content was determined with a diffusion chlorophyll meter (Konica Minolta, Langenhagen, Germany) according to established protocols [91,92]. The LRWC was calculated based on the difference of fresh and dry (3 d at 80 °C) weights as follows: LRWC (%) = (fresh weight − dry weight) × 100%/fresh weight.

Lipid peroxidation products were determined as thiobarbituric acid reactive substances (MDA equivalents) by the thiobarbituric acid (TBA) method (Protocol S1-4) [93], whereas total ascorbate, ascorbic acid and dehydroascorbate contents were assessed by ascorbate oxidase method (Protocol S1-5) [77]. Determination of leaf ABA, JA, JA-Ile and OPDA contents relied on the procedure of Balcke et al. [94] and ACQUITY H-Class UPLC ultrahigh performance liquid chromatography system (Waters GmbH, Eschborn, Germany) coupled on-line to a QTRAP 6500 (AB Sciex, Darmstadt, Germany) triple quadrupole-linear ion trap instrument operating in negative multiple reaction monitoring mode under the settings summarized in Table S1-1.

### 4.4. Extraction and Derivatization of Carbonyl Compounds

For extraction and derivatization of plant-derived carbonyl compounds, the method of the Fedorova’s group, originally developed for primary fibroblasts and pooled blood plasma samples [31], was adjusted for the pea root nodule material. The frozen plant powder (20 mg) was re-suspended in 50 μL of methanol by vortexing (3 min). After this, 100 μL of 0.5% (*w*/*v*) ammonium acetate buffer (pH 4) were added (final pH 7.0), and the samples were sonicated during 15 min. Derivatization of reactive carbonyl compounds (RCCs) was accomplished by adding 10.5 μL of 100 mmol/L CHH stock solution and incubation for 1 h at 37 °C. For extraction of derivatized RCCs, 350 μL of methanol and 1250 μL methyl tert-butyl ether were added to the samples, vortexed during 10 s and incubated during 1 h at 4 °C under continuous shaking (450 rpm). Afterwards, 313 μL of water were supplemented to the samples and the mixtures were incubated during 10 min at 4 °C under continuous shaking (450 rpm). After centrifugation (10 min at 10,000× *g*), the upper phase was transferred to new polypropylene tubes, and the residues were extracted for the second time with the same solvent. The resulting upper phase was combined with the first portion and dried under reduced pressure. For LC-MS analysis, the residues were reconstituted in 20 μL of acetonitrile, and 80 μL of 0.1% (*v*/*v*) aq. formic acid (FA) were added. The samples were two-fold diluted with 20% (*v*/*v*) acetonitrile in 0.1% (*v*/*v*) aq. FA, centrifuged (3 min at 10,000× *g*) and analyzed within 24 h.

### 4.5. UHPLC-ESI-LIT-Orbitrap-MS

Derivatized carbonyl compounds dissolved in 20% (*v*/*v*) acetonitrile in 0.1% (*v*/*v*) aq. FA were separated at the flow rate of 0.4 mL/min on an Acquity UPLC BEH300 C4 column (2.1 × 100 mm, 1.7 μm particle size) using a Dionex UltiMate 3000 UHPLC system coupled on-line to an Orbitrap Elite mass spectrometer via a HESI source (Thermo Fisher Scientific, Bremen, Germany). The eluents A and B were 0.1% (*v*/*v*) aq. FA and 0.1% (*v*/*v*) FA in acetonitrile, respectively. The derivatized RCCs were eluted with a linear gradient ramping from 20 to 95% B over 10 min. Afterwards, the column was washed with 95% eluent B for two min, and re-equilibrated at 20% eluent B for 6.5 min. The UHPLC-Orbitrap-MS analysis relied on data-dependent acquisition experiments performed in the positive ion mode, comprising a survey Orbitrap-MS scan and MS/MS scans for the three most abundant signals selected in the survey run. For this, a gas phase fractionation approach [32] was used, i.e., alternative runs were performed with survey scans acquired in the *m*/*z* ranges of 280–00, 395–500, 495–600, 595–700, 695–800, 795–900, 895–1000 or the whole mass range of 280–2000. For analysis, the samples were randomized and quality control runs, i.e., pooled methanol−methyl *tert*-butyl ether extracts obtained by combining 30 µL of all individual stressed and control samples, were injected (10 µL) throughout the whole complex batch every 12 runs (time increment of 255 min). The mass spectrometer settings and data-dependent acquisition parameters are summarized in the Supplementary Materials (Table S1-2).

### 4.6. Annotation and Relative Quantification of Individual RCCs

Derivatized RCCs were annotated by t_R_ and exact *m*/*z* values (mass accuracy within 3 ppm). For quantitative analysis, the peak areas in XIC (*m*/*z* ± 0.02) corresponding to the most abundant ions with signal intensities of more than 1 × 10^5^ counts were integrated in Xcalibur Quantitative analysis tool of the Xcalibur software (Thermo Fisher Scientific, Bremen, Germany).

### 4.7. Targeted MS/MS Analysis

The signals of RCC derivatives, which were not involved in fragmentation during data-dependent acquisition experiments, were selected for the targeted linear ion trap-MS/MS analysis with collision-induced dissociation at the normalized collision energy of 30%, activation frequency 0.25 Hz and isolation width of 2 Da.

### 4.8. Computational Correction of Analyte Degradation/Transformation

Assessment of analyte stability relied on quality controls, acquired as described above. Several runs of k-means clustering with Euclidian, dynamic time warping and dynamic time warping barycenter averaging [95] distance metrics suitable for time series analysis were performed to address the stability of RCCs derivates. Afterwards, regression analysis of the corresponding signal intensities in the quality controls was accomplished to obtain differential time patterns of degradation kinetics. After integration, peak areas were normalized by MinMax scaling to the range between −2 and 2. Using this strategy, all derivatized RCCs could be distributed in five clusters on the basis of their degradation kinetics. Based on these data, individual correction coefficients for signal intensities could be obtained for each analyte in control and stress samples based on specific time points at which the samples were analyzed. Applying the correction coefficients to the original signal intensities would eliminate the contribution of analyte degradation or/and solvent evaporation to the quantitative data. Stability analysis was performed in Python 3.7.4 using tslearn [96], numpy [97], scikit-learn [98], matplotlib [99] and seaborn [100] libraries. The individual steps of this protocol are explained in more detail in Protocol S1-1.

### 4.9. Statistical Analysis

To assess the statistical significance of the observed stress-related differences, the Student’s *t*-test for independent samples was applied. For this, normality of the distribution and the equality of the sample variances were assessed. The procedure was based on a visual examination of density plots (Figure S1-5) generated for the top 50 confident analytes. For the analytes confirmed as significantly regulated under drought conditions hierarchical clustering analysis was accomplished using the online resource MetaboAnalyst 4.0 (http://www.metaboanalyst.ca).

## 5. Conclusions

Drought dramatically affects productivity of legume plants, compromising stability and efficiency of legume-rhizobial symbiosis. At the experimental level, drought stress response, which is manifested by enhanced ROS production and oxidative stress along with essential rearrangements of plant metabolism, can be modeled by PEG-induced osmotic stress. Therefore, to get the first insight in the response of the root nodule carbonyl metabolome to water stress, we addressed here the effects of the PEG-induced osmotic stress with the intention to extend it further to physiological drought established in an appropriate soil-based stop watering model. As can be seen from our results, at the level of root nodule both oxidative stress and metabolic rearrangement are playing concretely, decelerating, and suppressing nitrogen fixation. Indeed, oxidation (most likely, by radical mechanism) of ALA to DOVA, yields a reactive carbonyl compound with a well-defined biological activity—inhibition of ALA dehydratase and blockage of porphyrin biosynthesis. This, in turn, results in disturbance of heme formation and biosynthesis of leghemoglobin in root nodules. This well-documented drought-related drop in leghemoglobin contents makes efficient oxygen buffering in nodules not possible anymore. This, in turn, results in suppression of nitrogenase activity. The described mechanism might be critical for re-direction of plant carbon flux from support of symbiosis to survival of the organism under stress conditions. However, the molecular basis of the DOVA effects is still to be revealed. Most likely, this mechanism relies on covalent modification of specific residues in the sequence of ALA dehydratase. Identification of these sites and related alterations is enzyme structure and activities need to be the matter of the future research.

## Figures and Tables

**Table 1:** Differentially abundant derivatized RCCs annotated in root nodules of *P. sativum* by reversed phase ultra-high-performance chromatography-tandem mass spectrometry (RP-UHPLC-MS/MS).

#	t_R_ (Min)	*m*/*z* [M + H]^+^ Observed	*m*/*z* [M + H]^+^ Calculated	Elemental Composition	Fragmentation Patterns (*m*/*z* (%))	Error (ppm)	Fold Change	Assignment
1 *	3.7	348.1555	348.1554	C_17_H_22_N_3_O_5_	216.1 (6), 261.1 (100), 262.1 (31), 276.1 (19), 301.2 (3), 303.2 (9.4), 318.1 (94), 319.2 (6), 330.1 (13), 331.2 (3)	−0.3	2.7↑	glyceraldehyde
2 *	6.2	388.1507	388.1503	C_19_H_22_N_3_O_6_	216.0 (7.1), 262.2 (18.6), 276.1 (5.7), 288.1 (7.1), 301.0 (6.4), 315.9 (2.9), 318.1 (13.6), 328.1 (9.3), 343.2 (7.9), 344.1 (15.7), 352.4 (15.0), 360.0 (4.6), 370.1 (100), 371.1 (20.0)	−1.0	2.1↑	4,5-dioxovaleric acid
3 *	6.6	288.1344	288.1344	C_15_H_18_N_3_O_3_	216.2 (7), 217.1 (18), 218.2 (40), 225.2 (12), 232.1 (100), 258.3 (9), 260.2 (40), 261.3 (65.7), 262.2 (89), 271.3 (6), 276.0 (12)	0.0	1.5↓	formaldehyde
4 *	7.9	316.1655	316.1656	C_17_H_22_N_3_O_3_	216.1 (18), 218.2 (58), 219.1 (24), 258.2 (100), 260.1 (42), 272.4 (47), 298.2 (67), 299.4 (76)	0.3	1.7↓	unknown *m*/*z* 316.17, propanal-related signal 1
5	8.2	372.1914	372.1917	C_20_H_26_N_3_O_4_	218.1 (0.05), 244.1 (30), 261.1 (0.5), 276.1 (16), 302.2 (0.6), 315.2 (0.25), 328.3 (0.1), 342.2 (0.3), 353.0 (2.5), 354.2 (100)	0.8	1.6↓	4-hydroxy-5-hexenal, isomer 1
6 *	8.3	316.1655	316.1656	C_17_H_22_N_3_O_3_	217.1 (9), 218.2 (3), 232.2 (14), 256.3 (16), 258.2 (100), 260.2 (25), 262.2 (21), 298.1 (20), 299.3 (38), 300.2 (29)	0.3	2.0↓	unknown *m*/*z* 316.17 propanal-related signal 2
7	8.3	398.2074	398.2073	C_22_H_28_N_3_O_4_	123.0 (3.3), 138.0 (0.5), 244.1 (16), 260.2 (0.7), 276.2 (1.4), 298.1 (0.22), 311.1 (0.35), 316.2 (0.3), 328.2 (0.37), 337.2 (2.2), 340.2 (0.4), 356.2 (1.1), 363.2 (1.8), 366.2 (1.2), 380.2 (100), 381.2 (15), 397.3 (0.6)	−0.3	1.6↓	hydroxy-2,4-octadienal
8	8.4	372.1917	372.1917	C_20_H_26_N_3_O_4_	216.2 (0.18), 244.1 (55), 261.3 (0.3), 262.3 (0.55), 276.1 (100), 300.3 (0.15), 313.9 (0.1), 329.7 (0.17), 342.2 (1.8), 354.2 (2.4), 355.2 (0.35)	0.0	1.6↓	4-hydroxy-5-hexenal, isomer 2
9 *	8.5	328.1653	328.1656	C_18_H_22_N_3_O_3_	216.1 (32), 232.1 (8), 256.1 (2), 260.1 (100), 271.1 (2), 283.2 (10), 300.1 (18), 311.2 (6)	0.9	2.1↓	methacrolein
10	8.5	360.1915	360.1915	C_19_H_26_N_3_O_4_	216.1 (0.04), 232.3 (0.02), 244.1 (1.05), 261.2 (0.06), 276.1 (0.08), 297.1 (1.17), 302.2 (0.08), 314.1 (1.24), 315.2 (0.36), 327.0 (1.13), 328.2 (100)	0.6	1.8↓	3-hydroxy-pentanal
11 *	8.5	414.2021	414.2022	C_22_H_28_N_3_O_5_	139.1 (1), 276.1 (22), 288.2 (7), 302.2 (1) 316.1 (100), 317.1 (10), 340.2 (1), 354.2 (11), 380.2 (4), 396.2 (62)	0.2	2.2↓	8-oxo-4-hydroxy-6-octenal
12 *	8.5	426.2021	426.2022	C_23_H_28_N_3_O_5_	122.1 (0.5), 166.0 (0.5), 218.1 (0.5), 262.2 (3.3), 276.1 (11), 298.1 (0.5), 330.2 (1.4), 352.2 (1.4), 354.2 (7.6), 366.2 (2.4), 380.2 (100), 381.2 (22), 382.2 (45), 383.3 (11), 394.2 (1.4), 408.2 (40)	0.2	2.7↓	9-oxo-2,4-nonadienoic acid
13	8.8	444.2120	444.2127	C_23_H_30_N_3_O_6_	183.1 (0.15), 244.1 (44), 262.2 (0.52), 276.1 (100), 300.2 (0.3), 302.3 (0.4), 316.2 (5), 332.3 (0.5), 346.2 (0.15), 356.1 (0.3), 368.1 (0.4), 370.2 (0.6), 384.2 (0.4), 408.2 (1.9), 426.3 (4.9)	1.6	2.1↓	9-oxo-2,6-dihydroxy-1-nonenal
14	8.8	472.2450	472.2439	C_25_H_34_N_3_O_6_	139.0 (11), 154.1 (0.2), 175.1 (0.3), 193.1 (1.6), 212.1 (1.8), 244.1 (18), 261.0 (0.8), 276.1 (8), 316.2 (4.4), 330.3 (0.4), 352.2 (0.4), 380.3 (0.4), 394.3 (1.2), 396.2 (2), 418.2 (3), 427.2 (4.7), 436.2 (12.6), 437.3 (3.2), 454.2 (100), 455.2 (11.8)	−2.3	3.8↓	4-hydroxy-11-oxo-2-undecenoic acid
15 *	8.9	330.1811	330.1812	C_18_H_24_N_3_O_3_	216.1 (1.2), 218.1 (2.4), 232.2 (1.2), 258.3 (0.5), 260.1 (3.6), 261.1 (31), 262.1 (3.1), 276.3 (0.5), 287.2 (2.4), 301.2 (1.2), 302.3 (1.1), 312.1 (7.4), 313.2 (100), 314.1 (3.1)	0.3	1.6↓	butanal
16	9.4	388.2226	388.2229	C_21_H_30_N_3_O_4_	216.1 (0.9), 232.1 (0.7), 244.1 (10), 261.2 (0.5), 273.2 (0.3), 276.2 (0.3), 302.2 (3.4), 316.2 (0.2), 332.2 (0.6), 344.1 (0.4), 356.2 (100), 370.3 (6), 371.2 (28)	0.8	2.0↓	7-hydroxy-heptanal
17 *	9.9	288.1346	288.1344	C_15_H_18_N_3_O_3_	178.2 (11), 202.2 (14), 216.1 (32), 217.1 (19), 225.2 (16), 230.2 (32), 232.2 (63), 260.1 (100), 261.1 (46), 262.2 (63), 270.1 (35)	−0.7	5.3↓	Unknown *m*/*z* 288.13, formaldehyde-related signal 1
18	10.1	496.2807	496.2802	C_28_H_38_N_3_O_5_	218.2 (2.1), 244.1 (100), 261.2 (1.1), 276.2 (10.5), 316.1 (57.9), 340.3 (2.1), 351.3 (3.7), 368.1 (2.1), 380.2 (51.6), 381.2 (10.5), 396.2 (6.3), 436.2 (12.1), 438.3 (24.2), 460.1 (4.2), 478.3 (34.7)	−1.0	1.7↑	unknown, *m*/*z* 496.28, signal 4
19 *	10.2	368.1964	368.1968	C_21_H_26_N_3_O_3_	108.0 (100), 201.3 (0.5), 218.2 (8.4), 261.1 (16.8), 276.1 (18.9), 302.1 (9.5), 324.3 (5.8), 333.3 (3.7), 339.0 (1.1), 348.2 (4.2), 350.3 (13.2), 351.3 (17.9), 352.2 (8.9)	1.1	1.7↓	2,4-heptadienal
20 *	10.3	444.2495	444.2490	C_24_H_34_N_3_O_5_	184.1 (20.9), 218.1 (3.5), 262.1 (4.0), 276.1 (4.7), 316.1 (4.0), 346.1 (1.2), 356.2 (2.3), 370.3 (1.2), 384.2 (17.4), 401.2 (4.7), 412.2 (100), 425.3 (20.9), 426.2 (15.1), 427.2 (31.4)	−1.1	1.9↓	10-oxo-4-hydroxy-decanal
21 *	11.0	288.1344	288.1344	C_15_H_18_N_3_O_3_	176.1 (9), 177.1 (19), 178.4 (8), 190.2 (8), 204.3 (13), 216.2 (50), 229.3 (9), 231.0 (20), 232.1 (75), 233.1 (28), 260.3 (66), 261.2 (91), 262.2 (100), 271.1 (31)	0.0	2.1↓	Unknown *m*/*z* 288.13, formaldehyde-related signal 2
22	13.5	758.5685	758.5667	C_40_H_79_N_4_O_7_P	244.1 (9), 281.1 (3), 307.3 (3), 325.2 (12), 327.2 (14), 353.3 (4), 387.3 (4), 423.3 (6), 453.2 (22), 455.3 (30), 475.2 (63), 477.2 (100), 478.2 (20), 479.3 (5), 501.4 (4), 529.5 (3), 573.5 (3), 601.5 (24), 603.6 (70), 611.5 (3), 629.6 (40), 646.6 (13), 671.6 (10), 699.6 (20), 701.6 (11), 713.5 (6), 723.6 (8), 739.6 (13), 740.6 (30), 741.5 (22), 759.5 (46)	−2.4	4.4↓	unknown, *m*/*z* 758.57, signal 2

* in these compounds the intensity of fragment ion with *m*/*z* 244.1 is not counted because of too high intensity of signal.

## Data Availability

Not applicable.

## Supplementary Materials

The following are available online at https://www.mdpi.com/article/10.3390/ijms23052726/s1.

## Abbreviations

ABA	abscisic acid
ALA	δ-aminolevulinic acid
CHH	7-(diethylamino)coumarin-3-carbohydrazide
DOVA	4,5-dioxovaleric acid
ESI	electrospray ionization
FA	formic acid
GAP	glyceraldehyde-3-phosphate
GAPDH	glyceraldehyde-3-phosphate dehydrogenase
HESI	heated electrospray ionization
HPLC	high performance liquid chromatography
HSA	human serum albumin
JA	jasmonic acid
JA-Ile	jasmonyl isoleucine conjugate
LC-MS	liquid chromatography-mass spectrometry
LC-MS/MS	liquid chromatography-tandem mass-spectrometry
LRWC	leaf relative water content
MDA	malondialdehyde
MS/MS	tandem mass spectrometry
NAD+	nicotinamide adenine dinucleotide
NADPH	nicotinamide adenine dinucleotide phosphate
OPDA	12-oxophytodienic acid
PEG	polyethylene glycol
RCCs	reactive carbonyl compounds
RNS	reactive nitrogen species
ROS	reactive oxygen species
RP-UHPLC-MS/MS	reverse phase ultra-high performance liquid chromatography-tandem mass spectrometry
RSD	relative standard deviation
SPE	solid phase extraction
TBA	thiobarbituric acid
TBARS	thiobarbituric acid reactive substances
UHPLC-ESI-LIT-Orbitrap-MS	ultra-high performance liquid chromatography—electrospray ionization—linear ion trap-Orbitrap mass spectrometry
t_R_	retention time
XIC	extracted ion chromatogram
