# Supplementary material for: Dynamics of Reactive Carbonyl Species in Pea Root Nodules in Response to Polyethylene Glycol (PEG)-Induced Osmotic Stress
Source: Int J Mol Sci. 2022 Mar 1;23(5):2726. doi: 10.3390/ijms23052726 (PMC8910736; doi:10.3390/ijms23052726)
Supplement: Supplementary file 1 [file ijms-23-02726-s001.zip › Supplementary information/211011_Soboleva_et_al_Supplementary information_1.pdf]

Dynamics of reactive carbonyl species in pea root nodules in response to osmotic stress – a new metabolic bridge to drought-related suppression of symbiotic efficiency

Alena Soboleva,[1,2*] Nadezhda Frolova,[3] Kseniia Bureiko,[1,2,4] Julia Shumilina,[1,2] Gerd U. Balcke,[5] Vladimir A. Zhukov,[6] Igor A. Tikhonovich,[6,7] Andrej Frolov,[1,2*]

Supplementary information 1

[1]Department of Bioorganic Chemistry, Leibniz Institute of Plant Biochemistry, [2]Department of Biochemistry, St. Petersburg State University, [3]Department of Plant Physiology and Biochemistry, St. Petersburg State University, [4]Institute of Biomedicine, University of Eastern Finland, Kuopio, Finland, [5]Department of Metabolic and Cell Biology, Leibniz Institute of Plant Biochemistry, [6]All-Russia Research Institute for Agricultural Microbiology and [7]Department of Genetics and Biotechnology, St. Petersburg State University

*Corresponding authors:

Mrs. Alena Soboleva
Leibniz Institute of Plant Biochemistry
Department of Bioorganic Chemistry
Weinberg 3
06120, Halle (Saale), Germany
Tel: +49 (0) 345 5582 1367
Fax: +49 (0) 345 5582 1359
E-mail: asoboleva@ipb-halle.de

Dr. Andrej Frolov
Leibniz Institute of Plant Biochemistry
Department of Bioorganic Chemistry
Weinberg 3
06120, Halle (Saale), Germany
Tel: +49 (0) 345 5582 1350
Fax: +49 (0) 345 5582 1359
Email: afrolov@ipb-halle.de

Directory

Protocol S1-1 Correction of analyte intensity data for derivative stability.. S1-3

Protocol S1-2 Determination of malondialdehyde (MDA) contents ……………….... S1-5

Protocol S1-3 Quantification of ascorbic acid and dehydroascorbate ……………... S1-6

Table S1-1 The conditions of UHPLC separation and the settings for ESI-QqQ-MS/MS………………………….……………….. S1-7

Table S1-2 Instrument settings applied for Orbitrap-LIT-MS experiments….. ..…. S1-11

Figure S1-1 Cultivation of pea (*P. sativum*) plants………………………............ S1-13

Figure S1-2 Physiological and biochemical characterization of pea (*P. sativum*) plant response to osmotic stress……………………………...……………………..…. S1-14

Figure S1-3 Clusterization of stability time curves of individual CHH derivatives of RCCs by characteristic kinetics profiles………………………………………...... S1-15

Figure S1-4 Types of stability profiles/degradation kinetics observed for CHH derivatives of RCCs…………………………………………………………………….... S1-16

Figure S1-5 Hierarchical clustering analysis with heatmap representation generated without application of correction for the changes in analyte abundance within the analysis time…………………………………………………………………………..... S-17

Figure S1-6 Fragmentation patterns of the CHH derivatives of 4,5-dioxovaleric acid and glyceraldehyde……..……………………………….……………………….. S1-18

Figure S1-7 Tandem mass spectrum of the unknown CHH derivative isomer 2 (*m/z* 758.5685) S1-19

References…………………..…………………………………………..…. S1-20

Protocols

Protocol S1-1 Correction of analyte intensity data for derivative stability

1. Data uploading

Using the *pandas* Python package, the matrix for pools (quality control, QC, designed as a pool of all experimental and control samples), control, and experimental samples with matched intensities for 194 compounds was uploaded as a *pandas DataFrame* from the csv file. Each sample was assigned to a certain time based on the timing of the instrumental sequence queue. The sequence queue was timed by QC, thereby the first QC served as the 0 point.

2. Clustering

Only QCs were used for clustering analysis of analyte kinetics behavior. It is important to note that the K-means clustering algorithm operates with the distances between individual features. Thereby, obviously, the differences in distances between high- and low-abundant signals will contribute more to the result, than the differences in degradation kinetics. This would result in clusters reflecting individual intensity-based variations rather than kinetics-based ones. This situation can be avoided by reduction of variables to the same scale for all analytes, i.e. by introduction a scaling procedure. This solution was implemented here by means of *Time Series Scaler MinMax* and *Time Serie sScaler Mean Variance* from the *tslean* package. The first approach yielded better results (in terms of significance of cluster definition) in first trials and was used, therefore, in all further analyses.

Furthermore, because the distance is not readily defined for time series data, it is important to consider utilizing variants of standard algorithms with alternative distance metrics suited for time series analysis, even if we have a small number of time points in our case study. For clustering TimeSeriesKMeans from tslearn package was used with different distance metrics

(Euclidian, Dynamic Time Warping (DTW) and DTW Barycenter Averaging (DBA)) and different number of clusters. Produced clusters appeared to be quite similar. Manual verification of the results obtained with all individual features allowed choosing the distance metric and number of clusters that produce the most relevant results for this task.

3. Regression analysis

Only pooled QC samples were used for linear regression analysis. Based on statistical analysis, the stability correction was applied to the cluster with the greatest RSDs and the most pronounced changes in compound abundance, corresponding to a substantial drop in compound abundance. These compounds were clustered once more in order to choose the degree of polynomial features in linear regression models for distinct types of degradation kinetics and avoid overfitting. The coefficient of determination and root mean square deviation were used to evaluate and compare the models' performance. Regression analysis was used to fit a predictive model to the original observed intensities of single compounds, and it was carried out according to standard procedure using the *sklearn* Python package.

4. Correction

In this part, the resulting model is utilized to estimate compound intensities at time points in the control and stress samples when true values were not observed. These values are then used to determine the coefficients for stability correction in control and stress samples as ratio of predicted value to intensity at the start of the experiment. These coefficients are then multiplied by the measured values in the control and stress samples. Finally, dry weight normalization was applied to all compounds and the results were extracted for further statistical analysis.

Protocol S1-2 Determination of malondialdehyde (MDA) contents

Determination of malondialdehyde (MDA) contents relied on Chantseva et al [1]. In detail, approximately 25 mg of frozen grinded plant material were left on ice for 3 minutes, before addition of 300 µL 5% (w/v) trichloroacetic acid (TCA), vortexed for 30 s and centrifuged at 10000 g for 20 minutes at 4°C. 250 µL of supernatant were transferred in a new polypropylene tube, and 1000 µL of thiobarbituric acid (TBA) reagent (0.5 % w/v TBA in 20% TCA) were added. The mixture was incubated for 30 min in boiling water bath (95°C). Afterwards, the mixture was cooled on ice to stop the reaction, centrifuged at 1900 g for 10 minutes at 4°C and 1 ml of colored supernatant was used to measure the absorbance at 532 nm against the proper blank (250 µL 5% w/v TCA and 750 µL TBA reagent). The non-specific absorbance at 600 nm was subtracted from the absorbance acquired at 532 nm. The contents of MDA equivalents were calculated with $\varepsilon = 155\ mM^{-1}cm^{-1}$

Protocol S1-3 Quantification of ascorbic acid and dehydroascorbate

Ascorbic and dehydroascorbic acids (Asc and DHA, respectively) were quantified in leaf tissue using an adaption of the method described by Frolov and co-workers [2]. In detail, 50 mg of frozen thallus material were left on ice for 3 min before 0.5 mL of ice-cold $HClO_4$ (2.5 mol/L) was added. The suspensions were vortexed for 30 s and centrifuged (10 min, 10,000 g, 4°C). The supernatants were transferred to new polypropylene tubes, neutralized with saturated Na_2CO_3 solution and diluted 10-fold with 0.1 mol/L sodium phosphate buffer (pH 5.6). For determination of ascorbic acid, 500 µL of the diluted extract was placed in a quartz cuvette and the absorbance at 265 nm was recorded (UV spectrophotometer, Beckman Coulter Deutschland, Krefeld, Germany) before 1 U of ascorbate oxidase (i.e. 1 µL in 4 mmol/L sodium phosphate buffer, pH 5.6) was added, and absorbance was recorded at the same wavelength after 2 min incubation at room temperature (RT). Total ascorbate was quantified after reduction of diluted extract with DTT (3 µL of 3 mol/L solution) for 1 min on ice at the same wavelength. Dehydroascorbic acid was calculated as the difference between the total ascorbate and ascorbic acid contents.

Tables

Table S1-1 The conditions of ultrahigh performance liquid chromatographic (UHPLC) separation and the settings for electrospray ionization-triple quadrupole-tandem mass spectrometry (ESI-QqQ-MS/MS) used for analysis of abscisic acid (ABA), jasmonic acid (JA), its isoleucine conjugate (JA-Ile) and 12-oxophytodienic acid (OPDA) in methanolic extracts of pea (*Pisum sativum* L., cultivar SGE) seeds with Waters ACQUITY UPLC H-Class UPLC System (Waters GmbH, Eschborn, Germany) coupled online to a hybrid triple quadrupole-linear ion trap mass spectrometer (QqLIT) AB Sciex QTRAP 6500 (AB Sciex, Darmstadt, Germany).

Chromatography

**ACQUITY Sample Manager (SM)**	
Injection mode	Partial Loop
Injection volume	5 µL
Weak wash solvent	0.3 mmol/L aq. ammonium formate
Weak wash volume	1200 µL
Strong wash solvent	Acetonitrile
Strong wash volume	600 µL
Target sample temperature	5.0 C
Needle overfill flush	Automatic
**Column conditions**	
Separation column	EC 150/2 NUCLEOSHELL RP 18<br>(150 x 2 mm, particle size 2.7 µm)
Target column temperature	40.0 C

ACQUITY Binary Solvent Manager (BSM)	
Eluent A	0.3 mmol/L aq. ammonium formate
Eluent B	Acetonitrile
Seal wash duration	5 min
Flow rate	0.4 mL/min
Elution program	5% eluent B isocratic – 2 min<br>gradient to 95% eluent B – 5.5 min<br>95% eluent B isocratic – 2 min<br>gradient to 5% eluent B – 0.01 min<br>5% eluent B isocratic – 1.99 min (re-equilibration)

Mass spectrometry

General	
Mass analyzer type	triple quadrupole-linear ion trap (QqLIT, QTRAP)
Ion source	Turbo Ion Spray®
Experiment type	multiple reaction monitoring (MRM)
Operatinon mode	negative
Cycle time (ms)	950
Pause between ranges (ms)	5.007
Settling time (s)	0
Duration	11 min
**Ion source settings**	
Nebulizer gas (psig)	60

Drying gas (psig)	70
Curtain gas (psig)	40
Ion spray voltage (kV)	-4.5
Ion source temperature (°C)	450
**MS/MS Setting**	
Fragmentation mode	CAD
MS/MS experiment type	MRM
Collision gas	nitrogen
Collision gas pressure	3 psig (medium)
Entrance potential (V)	-10.0
Scheduled MRM	enabled
Scheduled MRM type	basic
MRM detection window (s)	500
Target scan time (s)	1
Dwell time	adjusted by scheduled MRM algorithm
Q1 resolution	unit
Q3 resolution	unit
Declustering potential (DP, V)	compound-specific
Collision potential (CE, V)	compound-specific
Exit potential (CXP, V)	compound-specific

Analyte-specific settings

Analyte-specific combinations of Q1 and Q3 *m/z* ranges (transitions)						
**Analyte**	**t_R (min)**	**Q1 (*m/z*)**	**Q3 (*m/z*)**	**DP (V)**	**CE (V)**	**CXP (V)**

abscisic acid 01	4.8	263.0	152.9	-20.0	-16.0	-9.0
abscisic acid 02	4.8	263.0	219.0	-20.0	-18.0	-13.0
(d6) abscisic acid 01	4.8	269.0	159.0	-20.0	-16.0	-9.0
(d6) abscisic acid 02	4.8	269.0	225.0	-20.0	-18.0	-13.0

Table S1-2 Instrument settings applied for Orbitrap-LIT-MS experiments

Parameter	Setting
MS conditions	
Ionization mode	Positive
Mass analyzer	LIT-Orbitrap (FT-scan)
Ion spray voltage (IS)	4.5 kV
Nebulizer gas	35 psig
Auxillary gas	15 psig
Capillary temperature	300 °C
Mass to charge ratio (*m/z*) range	280 – 2000
Resolution	30000
MS/MS conditions	
Ionization mode	Positive
Mass analyzer	LIT (IT-scan)
Ion spray voltage (IS)	4.5 kV
Fragmentation	Collision activated dissociation
Isolation width	2 Da
Normalized collision energy	30%
Activation frequency	0.25 Hz
Activation time	10 ms
Default charge state	1
Dynamic exclusion repeat count	1

Dynamic exclusion duration	20 s
Dynamic repeat duration	30 s
Dynamic exclusion mass width	± 1.5 ppm
Mass ranges (*m/z*)	280-2000, 280-400, 395-500, 495-600, 595-700, 695-800, 795-900, 895-1000,

Figures

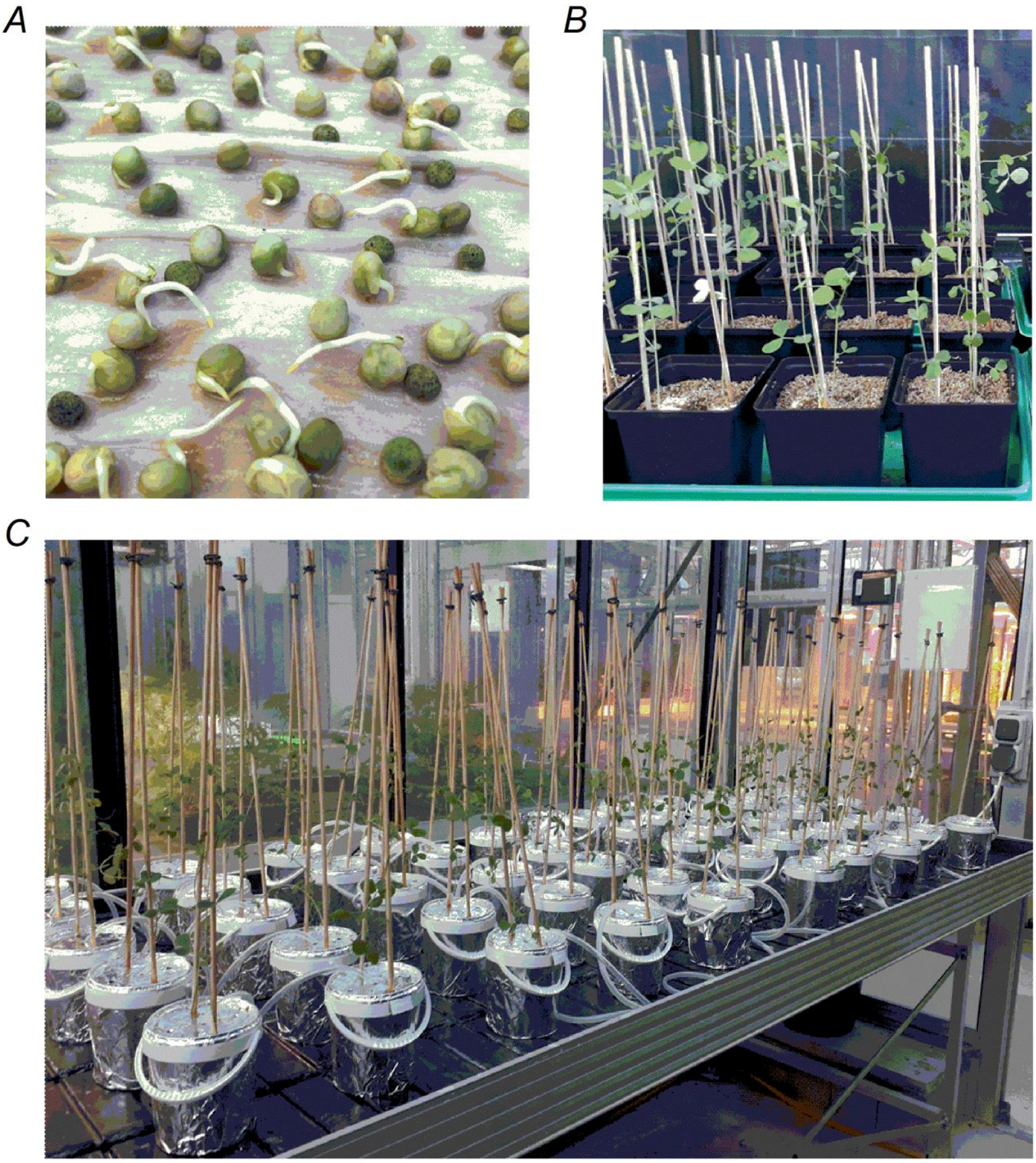

Figure S1-1 Cultivation of pea (*P. sativum*) plants: germinating seeds (two-day seedlings, A), two week-old plants before transfer to the hydroponic system (B), three week-old plants grown in the hydroponic system (C)

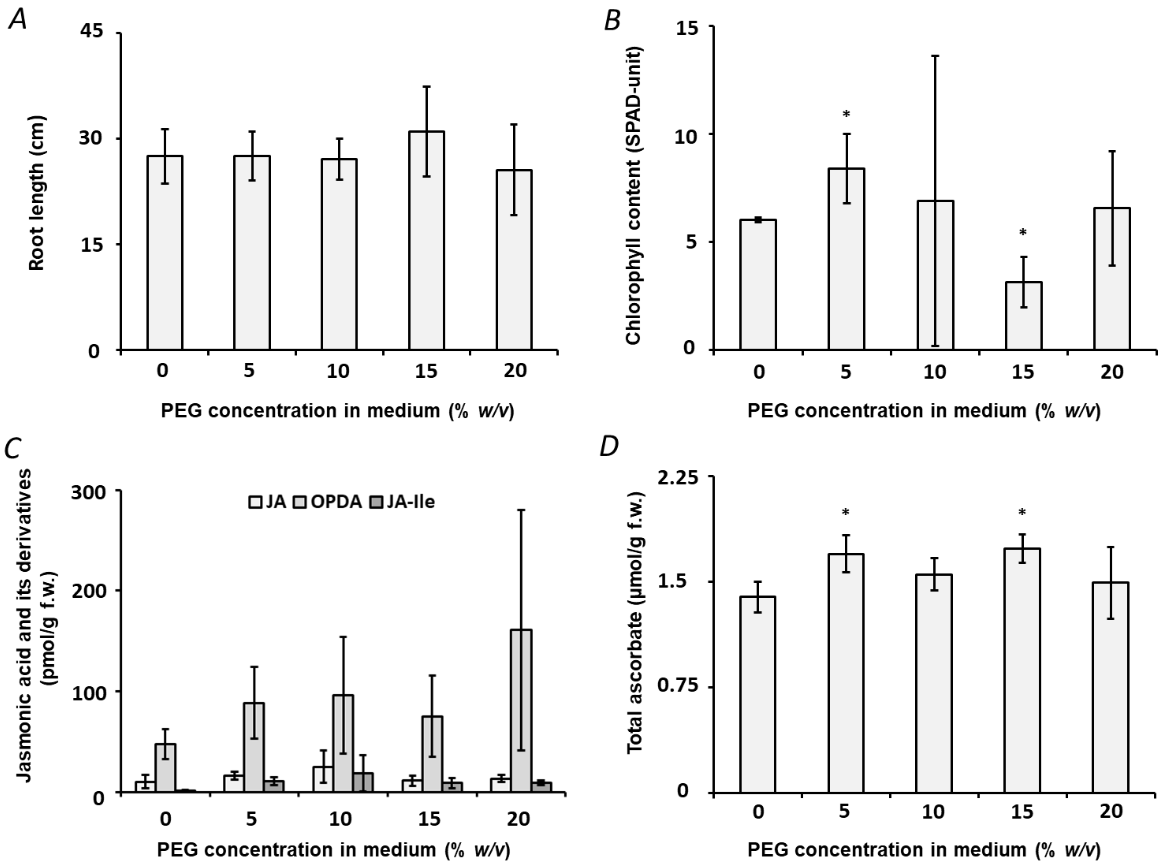

Figure S1-2 Physiological and biochemical characterization of pea (*P. sativum*) plant response to osmotic stress applied by transfer of two week-old plants to aqueous growth medium (with composition specified in the Material and method part) supplemented with 5, 10, 15 and 20% (w/v) PEG8000 for one week (controls were grown in PEG-free medium). The plant stress response was characterized at the end of the treatment period (7 days) with root length (A), chlorophyll content (B), content of jasmonic acid, its isoleucine conjugate (JA-Ile) and 12-oxophytodienic acid (OPDA) (C) and total ascorbate content (D). Statistically significant differences (t-test, $p \leq 0.05$) are marked with asterisk.

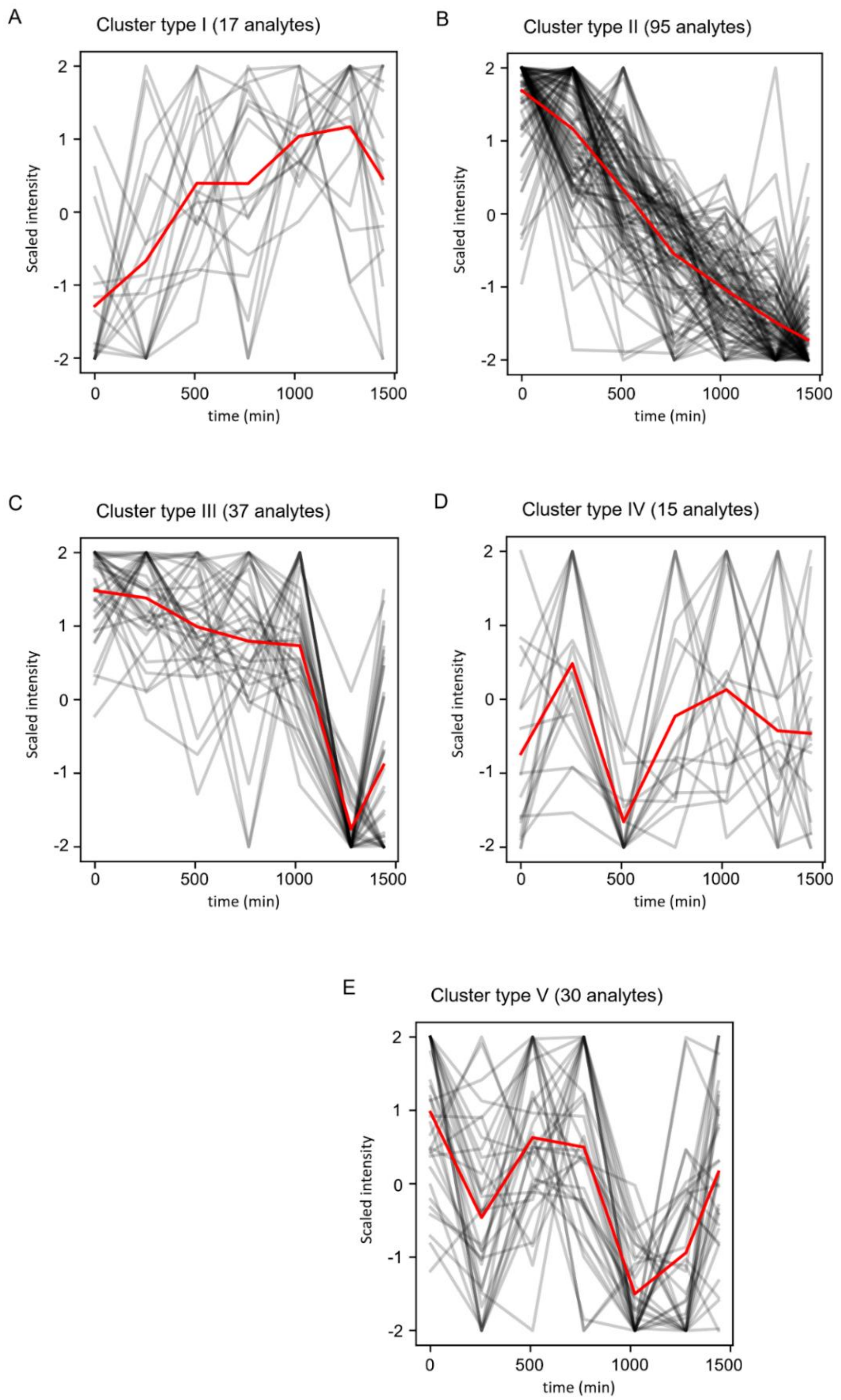

Figure S1-3 Cluster analysis of stability time curves of individual CHH derivatives of RCCs by characteristic kinetics profiles. Clustering relied on the standard K-means algorithm with Euclidian distance and pre-determined number of cluster 5 for preliminarily scaled dataset.

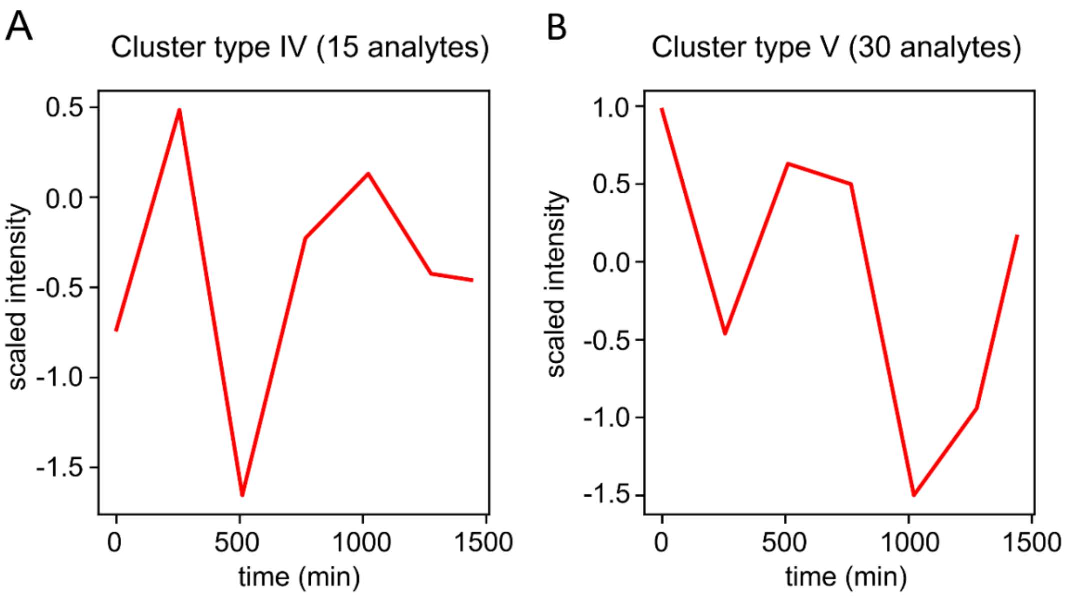

Figure S1-4 Types of stability profiles/degradation kinetics observed for CHH derivatives of RCCs. The centroids of the final clusters are defined as the average pattern from time curves of individual CHH derivatives of RCCs in a certain cluster produced using K-means clustering.

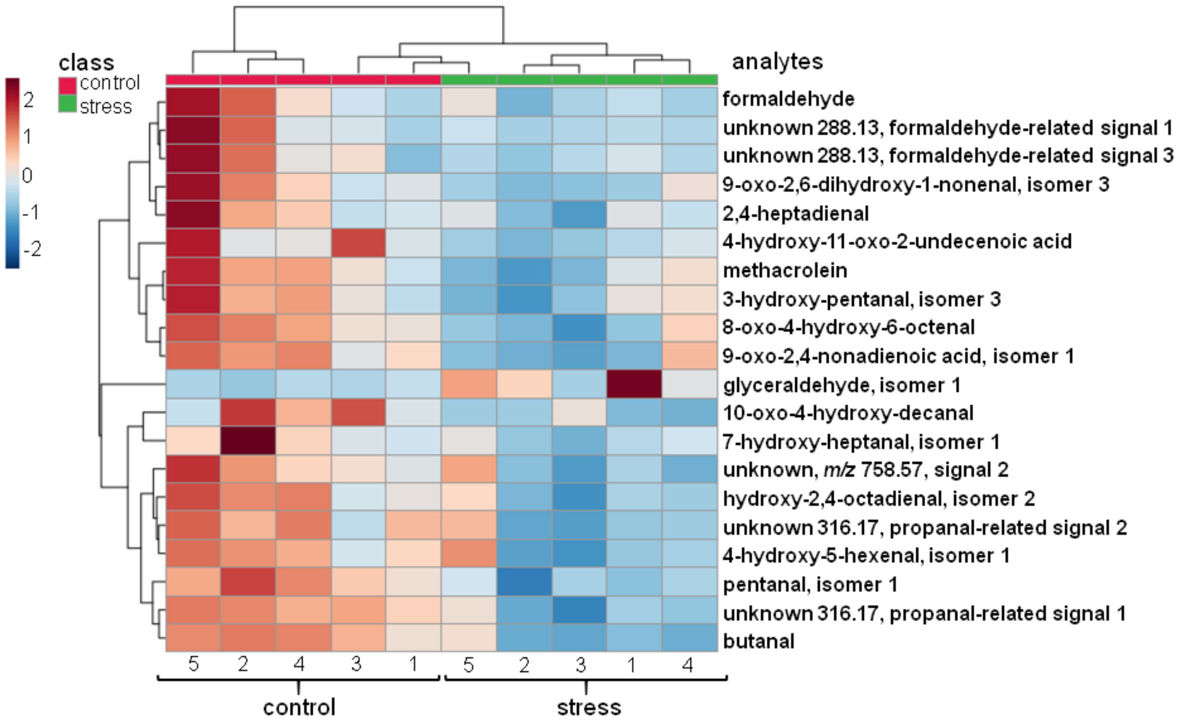

Figure S1-5 Hierarchical clustering with heatmap representation generated without application of correction for the changes in analyte abundance within the analysis time. The heatmap illustrates stress-dependent differential regulation of 20 RCCs in the root nodules of control and experimental pea (*P. sativum*) plants, treated with 10% (w/v) PEG8000 supplemented to the hydroponic growth medium for one week.

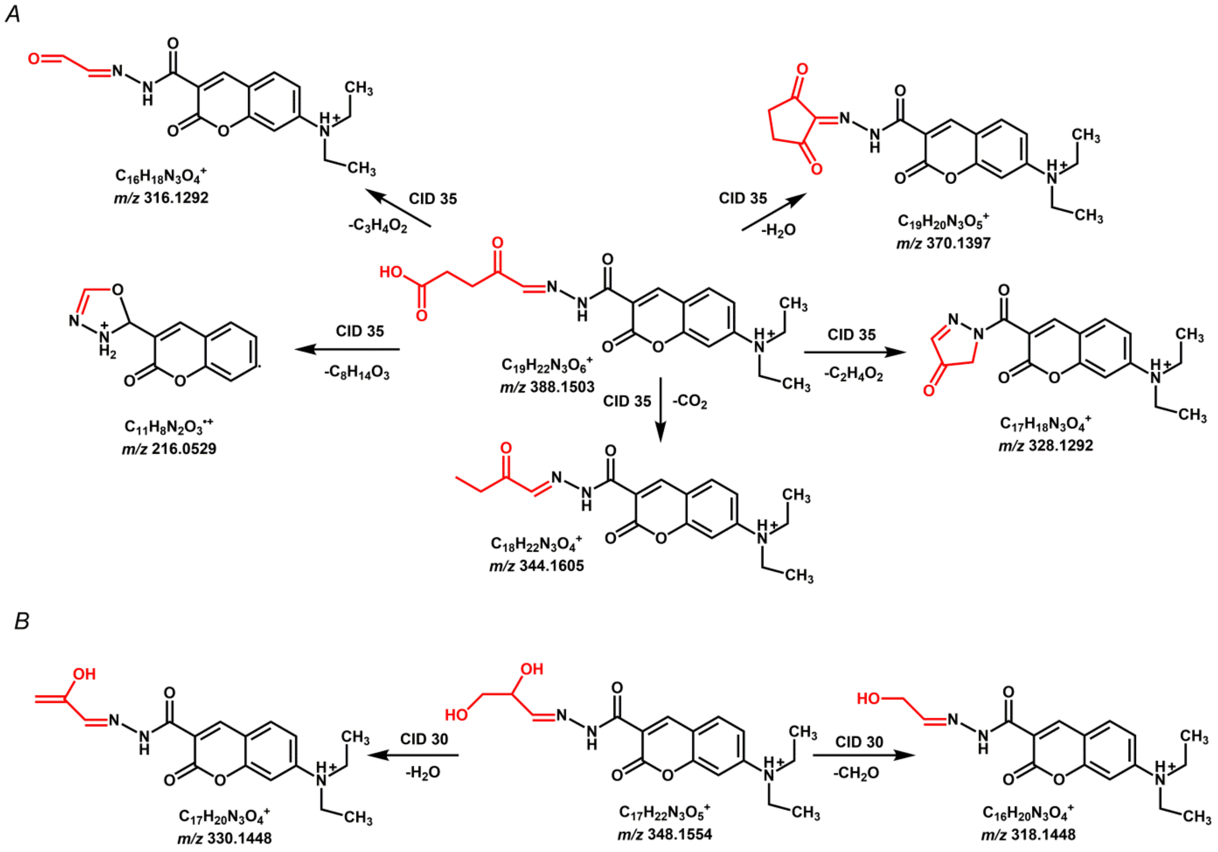

Figure S1-6 Fragmentation patterns of the CHH derivatives of 4,5-dioxovaleric acid at *m/z* 388.1503 (A) and glyceraldehyde at *m/z* 348.1554 (B) stress-dependently up-regulated in the root nodules of pea (*P. sativum*) plants, which were treated with 10 % (w/v) PEG8000 supplemented to the hydroponic growth medium for one week. The MS/MS analysis was accomplished in positive ion mode using collision-induced dissociation (CID) functionality of the linear ion trap (LIT) mass analyzer (30 % (A) and 35 % (B) normalized collision energy) of the Orbitrap Elite mass spectrometer. The part of the derivative corresponding to the RCC structure is marked red.

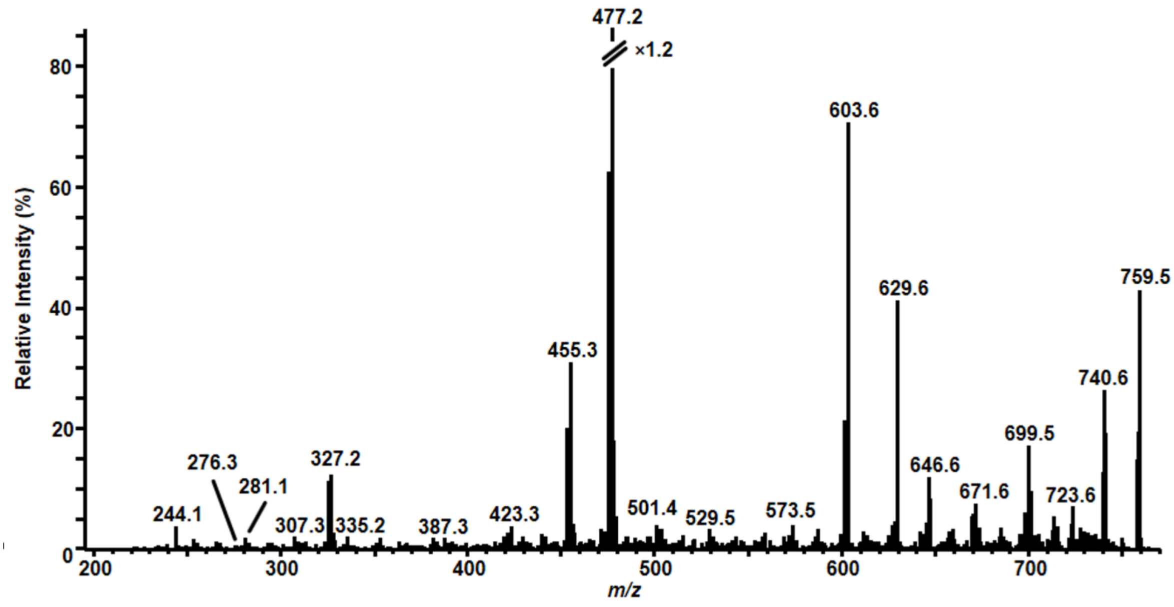

Figure S1-7 Tandem mass spectrum of the unknown CHH derivative isomer 2 (*m/z* 758.5685) differentially regulated in the root nodules of control and experimental pea (*P. sativum*) plants, treated with 10 % (w/v) PEG 8000 supplemented to the hydroponic growth medium for one week. The spectrum was acquired in positive ion mode using CID functionality of the LIT mass analyzer (35 % normalized collision energy) of the Orbitrap Elite mass spectrometer.

References

1. Chantseva V, Bilova T, Smolikova G, Frolov A, Medvedev S (2019) 3D-clinorotation induces specific alterations in metabolite profiles of germinating Brassica napus L. seeds. Biological Communications 64:55-74-55–74 . https://doi.org/10.21638/spbu03.2019.107

2. Frolov A, Bilova T, Paudel G, Berger R, Balcke GU, Birkemeyer C, Wessjohann LA (2017) Early responses of mature Arabidopsis thaliana plants to reduced water potential in the agar-based polyethylene glycol infusion drought model. J Plant Physiol 208:70–83 . https://doi.org/10.1016/j.jplph.2016.09.013
